# La_2_O_3_ Catalyzed C–C Coupling of Aryl Iodides and Boronic Acids

**DOI:** 10.5402/2012/814247

**Published:** 2012-10-01

**Authors:** Payal Malik, Debashis Chakraborty

**Affiliations:** Department of Chemistry, Indian Institute of Technology Madras, Chennai 600036, India

## Abstract

An efficient La_2_O_3_-catalyzed new route for the carbon-carbon bond formation in particular, symmetrical and unsymmetrical biphenyls has been developed, which proceeds through carbon-carbon coupling reaction of aryl iodides with boronic acids. The reaction provided the desired products in moderate-to-good yields with a wide range of functional group tolerance.

## 1. Introduction

The formation of new carbon-carbon bonds is of central importance in organic and medicinal chemistry [1, 2]. The development of new methods for carbon-carbon bond formation is a well-growing area in organic chemistry [3]. In the past decades, tremendous efforts have been devoted into the transition-metal catalyzed cross-coupling reactions [4]. The transition metals have played an important role in organic chemistry and this has led to the development of a large number of transition metal-catalyzed reactions for the formation of C–C and carbon-heteroatom bonds in organic synthesis [5, 6]. In the literature, a variety of nontransition and transition metals like palladium [7–9], copper [10, 11], iron [12], nickel [13, 14], cobalt [15], zinc [16], indium [17], solid supported catalyst [18], and metal nanoparticle [19] have been used for the coupling reactions. In fact palladium-catalyzed Suzuki-type cross-coupling reactions are very well explored and frequently used in organic synthesis and medicinal chemistry [1]. Organoborane and boronic acids have been utilised as arylating agent for the C–C bond formation [7–15]. 

Metal oxides represent one of the most important and widely used solid catalysts, either as active phases or as supports. The metal oxides are the largest family of catalysts in heterogeneous catalysis due to the acid-base and redox properties [20–23]. The outer electron configuration of the transition and noble group metals made them the most frequently used catalysts [24]. These metal oxides have been proved as efficient catalysts for the coupling reaction. Hell and coworkers reported copper-free Sonogashira reaction of alkynes and aryl halides by using Pd/MgLa mixed oxide [25]. Herein, we have developed a La_2_O_3_ catalyzed C–C coupling by using aryl halide and boronic acids.

## 2. Experimental Section

### 2.1. General Considerations

All the substrates used in this study were purchased from Aldrich and used as received. All the solvents were purchased from Ranchem, India and purified using standard methods. The products are characterized by recording ^1^H, ^13^C NMR, and ESI-MS by using Bruker Avance 400 MHz instrument and JEOL JMS GC-mate II instrument.

### 2.2. Typical Procedure for C–C Coupling Reaction

To a stirred solution of boronic acid (1 mmol) and La_2_O_3_ (10 mol%) in DMSO (3 mL) was added aryl iodides (1 mmol) followed by *trans*-1,2-diaminocyclohexane (10 mol%) and KO-*t-*Bu (2 equiv.). The reaction mixture was heated to 150°C and the progress of reaction was monitored by TLC. After completion, the reaction mixture was washed with EtOAc-H_2_O and the organic phase was separated and dried over Na_2_SO_4_. The EtOAc was evaporated, and the further purification was done by using column chromatography.

## 3. Result and Discussion

To optimize the reaction conditions, different bases and solvents were screened in the presence of La_2_O_3_ as catalyst for the C–C coupling reactions. For the initial studies, we chose phenyl iodide and phenyl boronic acid as model substrates and various ligands and bases were screened (Table 1). The results revealed that the *trans*-1,2-diamino cyclohexane (L2) was the best ligand for the coupling reaction. *N*,*N *′-dimethylethane-1,2-diamine (L4) was proved to be an effective ligand in the coupling reaction; in fact, the reaction took a little longer time to complete as compared to the L2 (entry 3 versus entry 16). On the basis of base optimization results, KO-*t*-Bu was found to be the best base among the rest of the bases which have been used for optimization (Table 1, entry 3). Among the different solvents, DMSO gave the best results (Table 1, entry 3). On the ground of optimization results, we concluded that phenyl iodide and phenyl boronic acid in combination of La_2_O_3_ (10 mol%), KO-*t*-Bu (2 equiv.), *trans*-1,2-diaminocyclohexane (L2) (10 mol%), and DMSO as solvent at 150°C is the most efficacious reaction condition.

After optimizing the reaction conditions, we have explored the substrate scope by carrying out the reaction with various aryl halides and the results are illustrated in Table 2. 

Alkyl substituted halide substrates (Table 2, entries 2–5) gave good yield as shown in Table 2. In case of phenyl boronic acid and phenyl iodide coupling reaction, the observed yield was good, since the homocoupled and coupled product is biphenyl. Electron donating substrates (Table 1, entries 2–5) provided biaryl in shorter reaction time as compared to the electron withdrawing substrates (Table 2, entries 2–5 versus entries 6–8). As a matter of fact, a small amount of homocoupled products were observed in the reaction mixtures. There is a report which discusses the formation of homo-coupled product from boronic acids under similar conditions [26]. To confirm this, we have performed the reaction without phenyl iodide and observed 5–15% of the homo-coupled product in the reaction mixture. 

## 4. Conclusion

In summary, we have developed an efficient La_2_O_3_-catalyzed new route for the carbon-carbon bond formation. The developed catalytic system shows the wide range substrate applicability and functional group tolerance. A small amount of homo-coupled aryls as side product was observed. 

## Supplementary Material

Supplementary material includes characterization data of all the products discussed in the Tables.

## Figures and Tables

**Table 1:** La_2_O_3_-catalyzed C–C coupling of aryl halides with phenyl boronic acid.

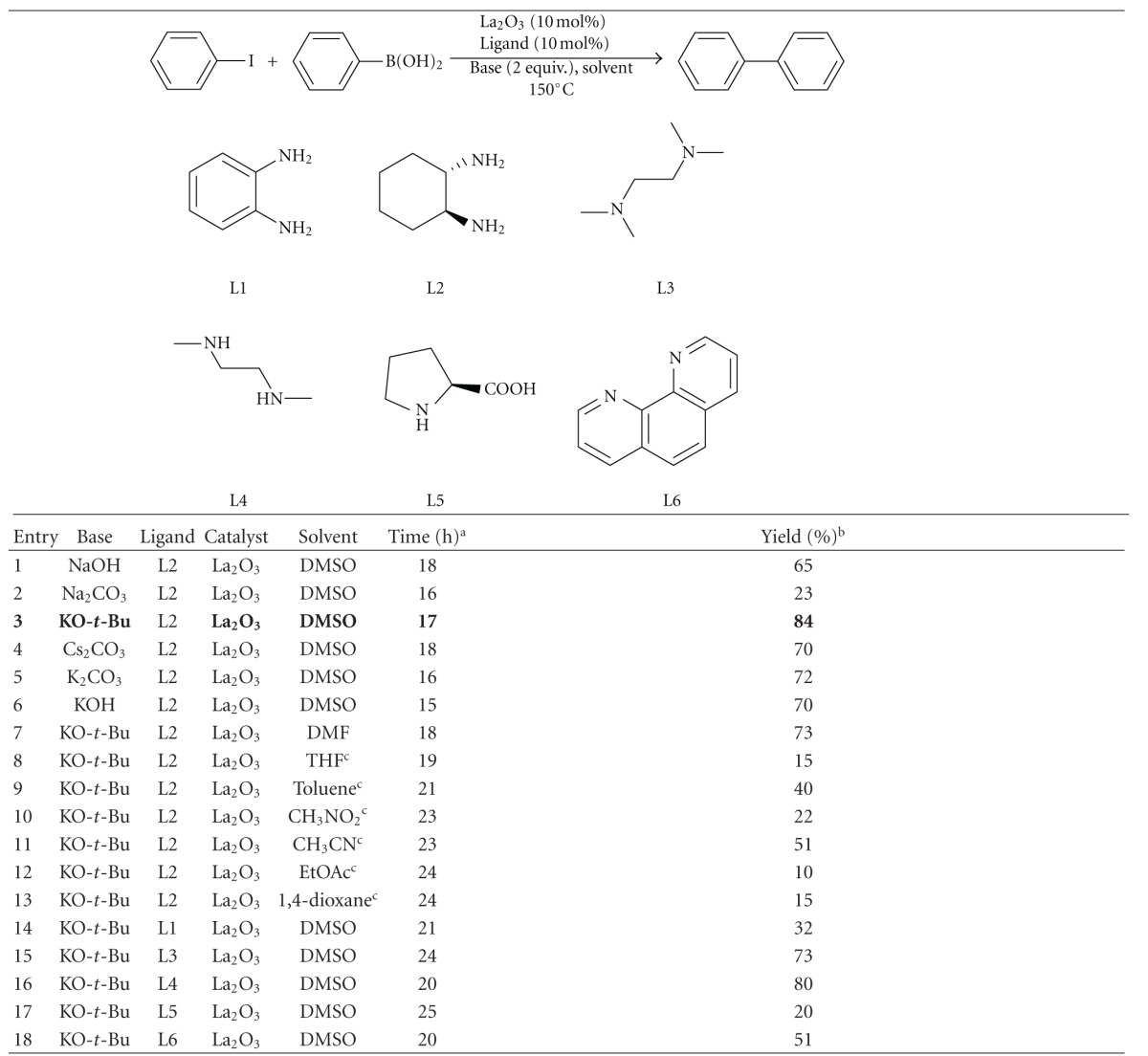

^a^Monitored using TLC. ^b^Isolated yield after column chromatography of the crude product. ^c^The reaction mixture was set to reflux.

**Table 2:** La_2_O_3_-catalyzed coupling of substituted phenyl iodide with substituted aryl boronic acids.

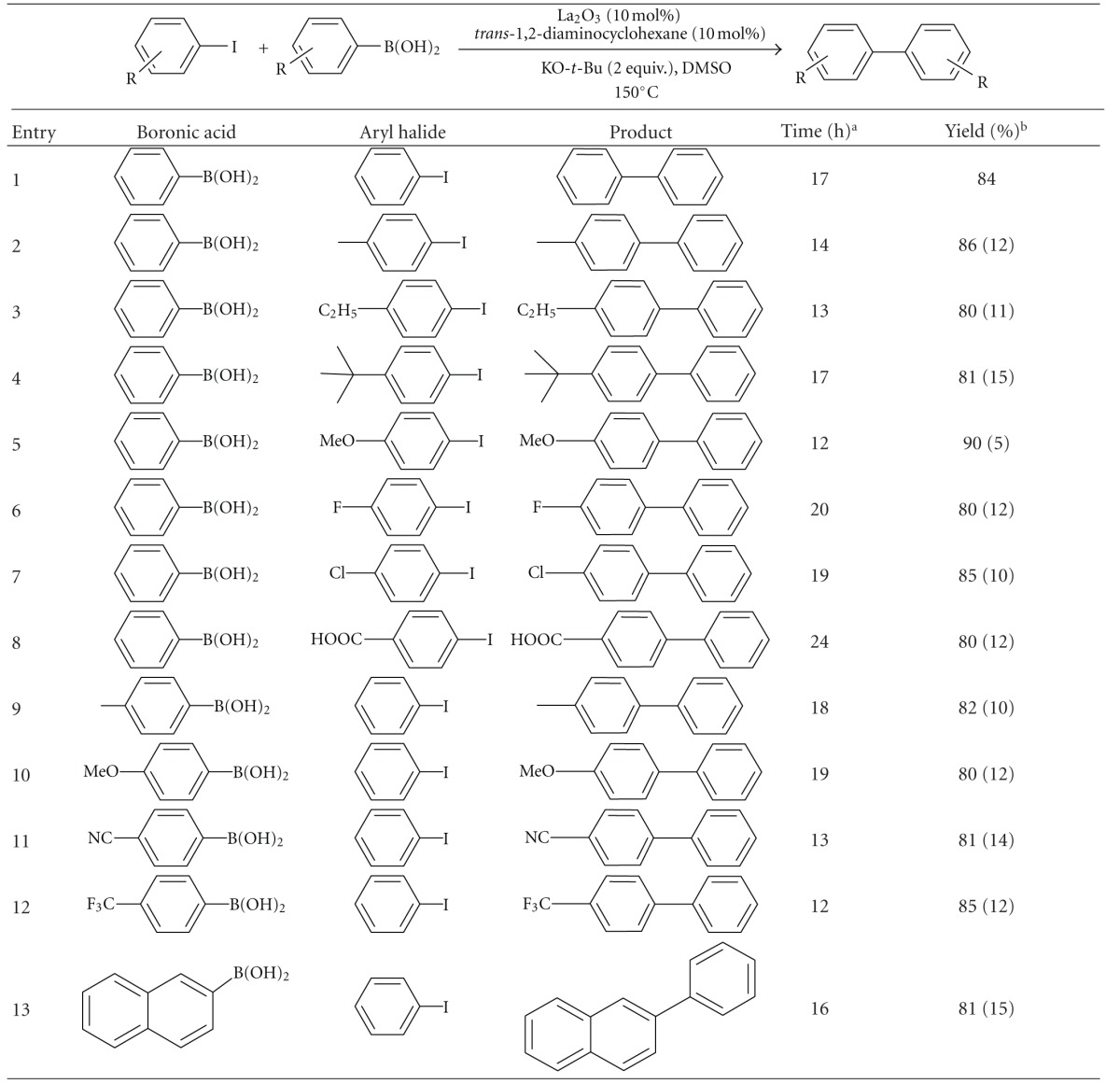

^a^Monitored using TLC. ^b^Isolated yield after column chromatography of the crude product. ^c^Yield in parenthesis is the homocoupling product from boronic acid.
