# Supplementary material for: La2O3 Catalyzed C–C Coupling of Aryl Iodides and Boronic Acids
Source: ISRN Org Chem. 2012 Oct 1;2012:814247. doi: 10.5402/2012/814247 (PMC3767364; doi:10.5402/2012/814247)
Supplement: Supplementary file 1 — Supplementary material includes characterization data of all the products discussed in the Tables. [file 814247.f1.pdf]

Supporting Information

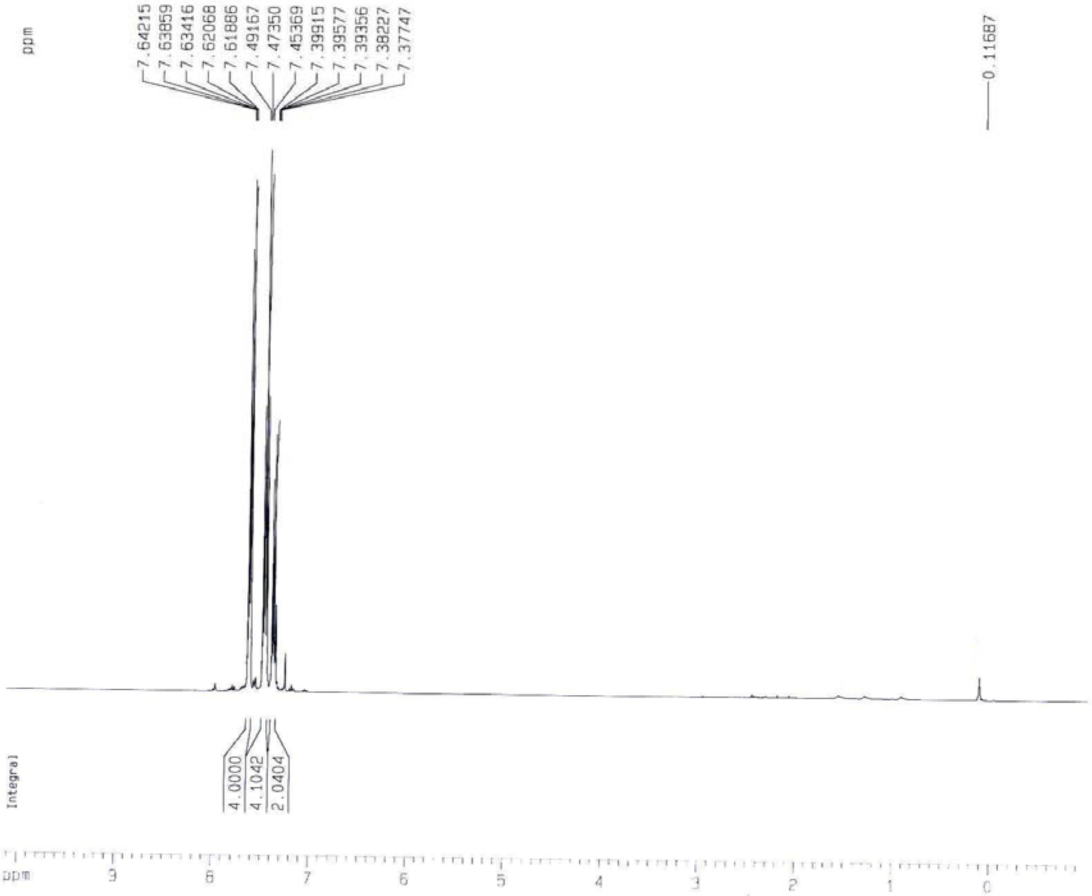

^{1}H-NMR spectrum of biphenyl (Table 1, Entry 1)

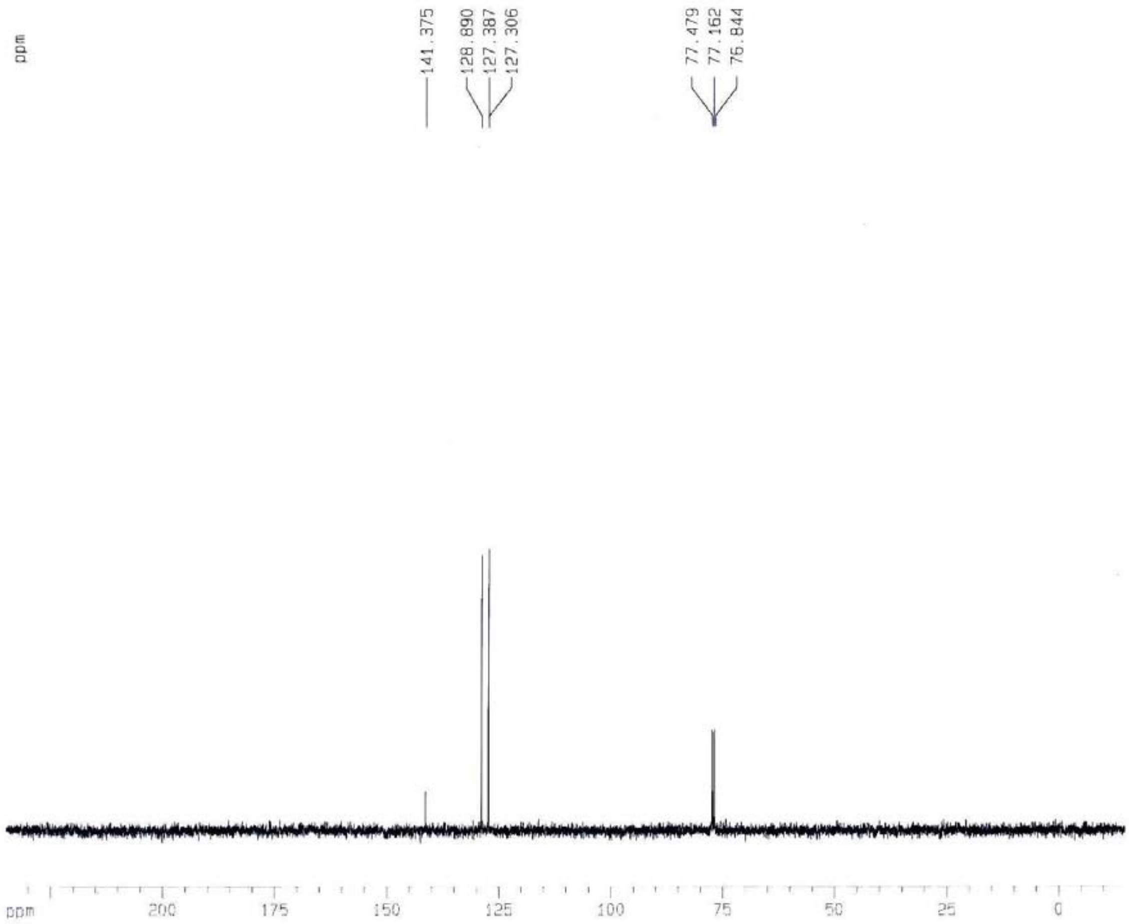

^{13}C-NMR spectrum of biphenyl (Table 1, Entry 1)

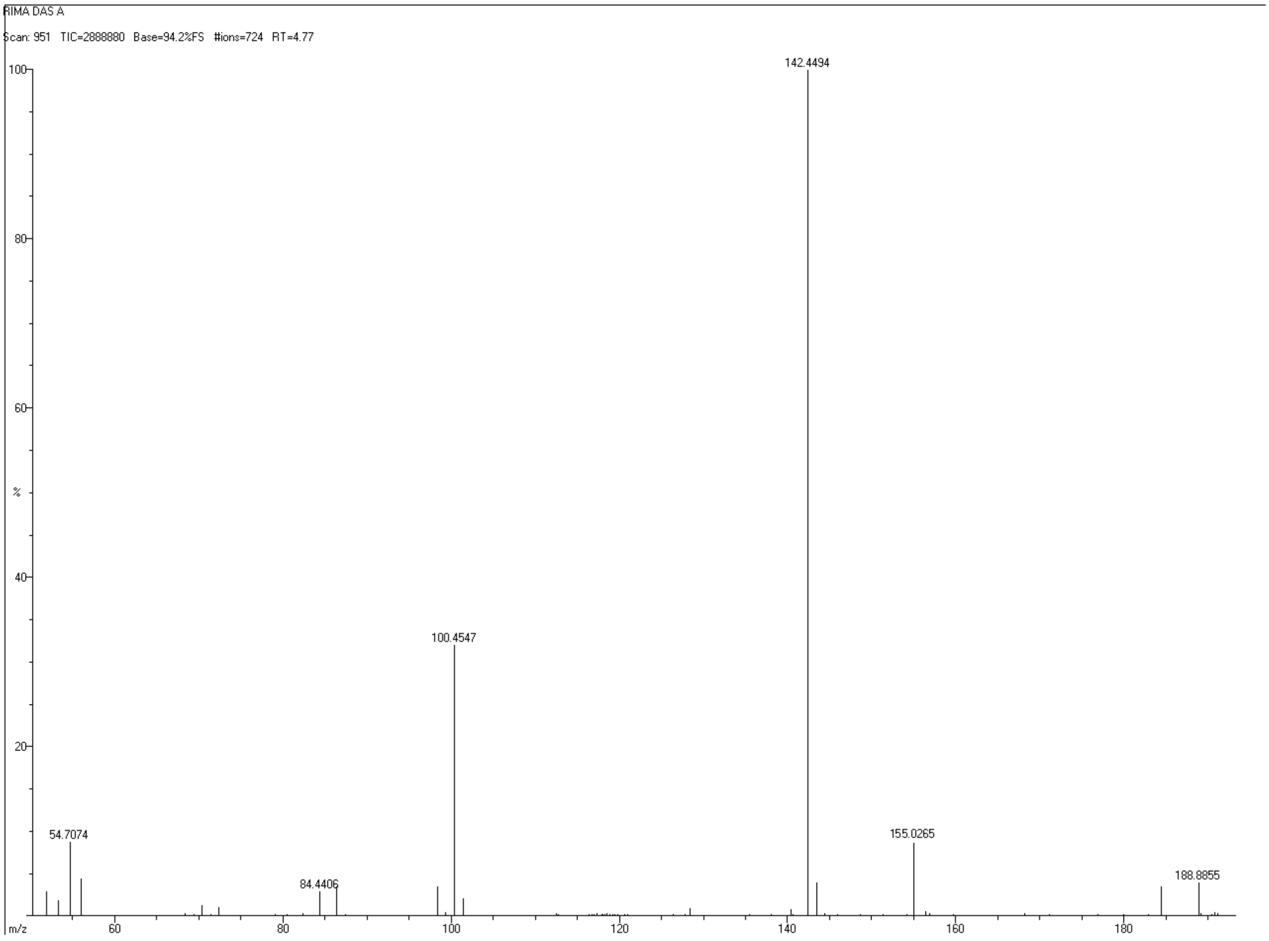

ESI-MS spectrum of biphenyl (Table 1, Entry 1)

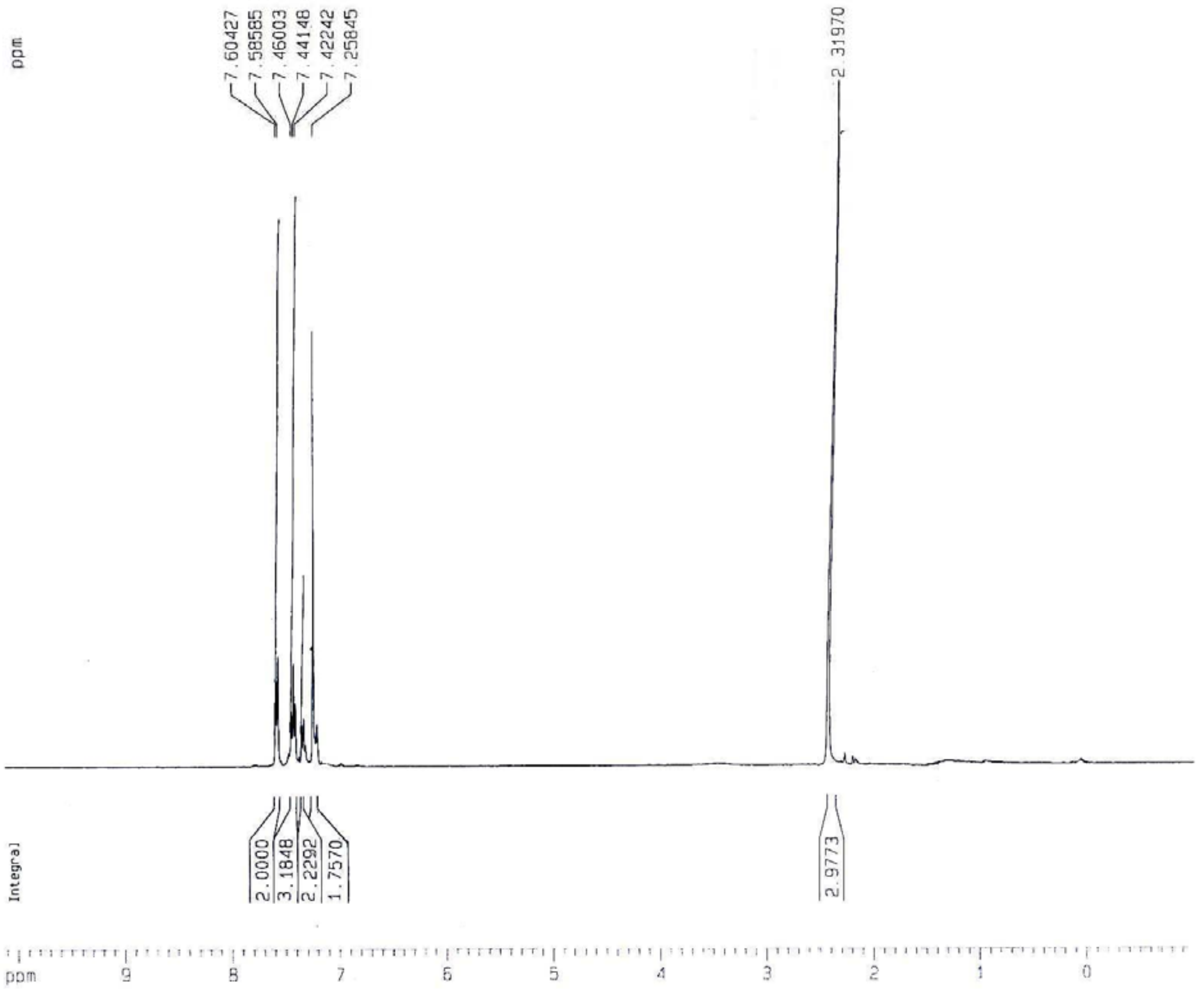

^{1}H-NMR spectrum of 4-methylbiphenyl (Table 1, Entry 2)

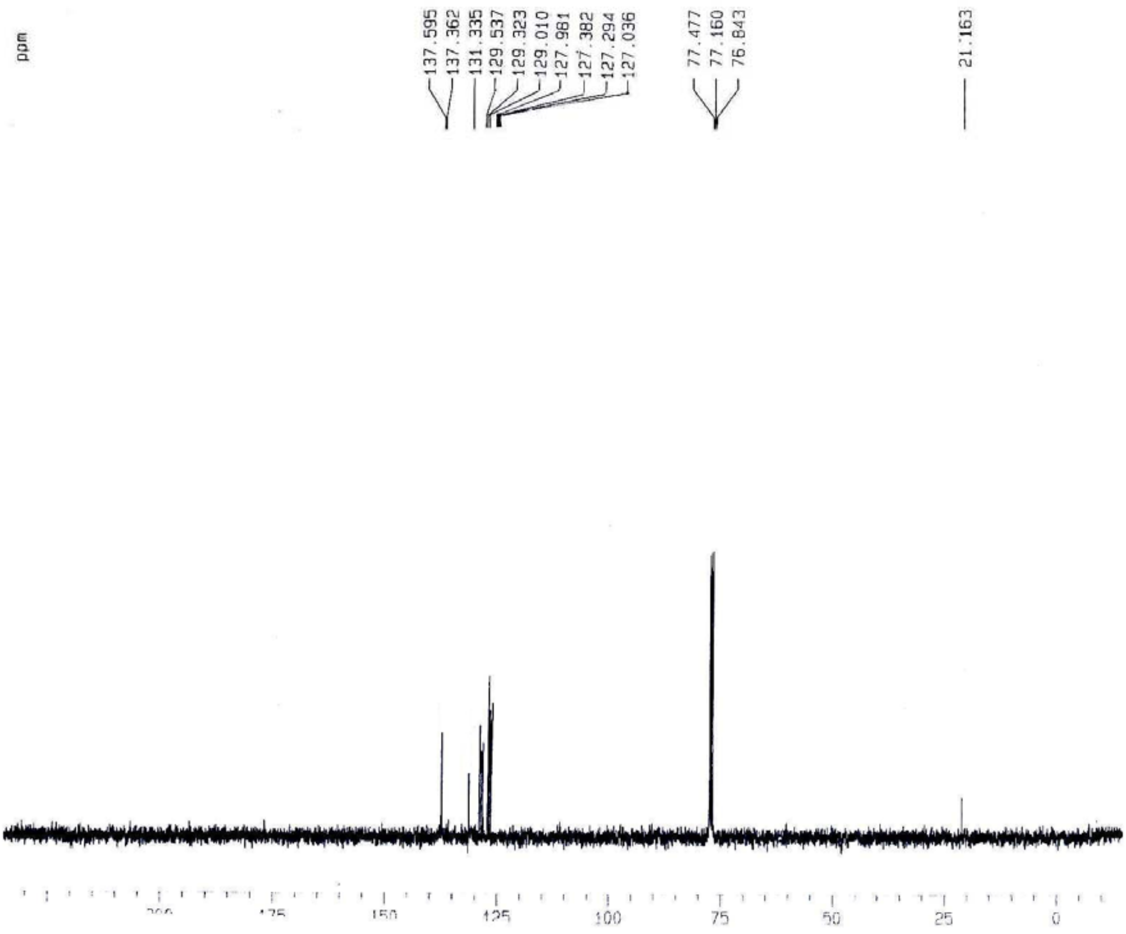

^{13}C-NMR spectrum of 4-methylbiphenyl (Table 1, Entry 2)

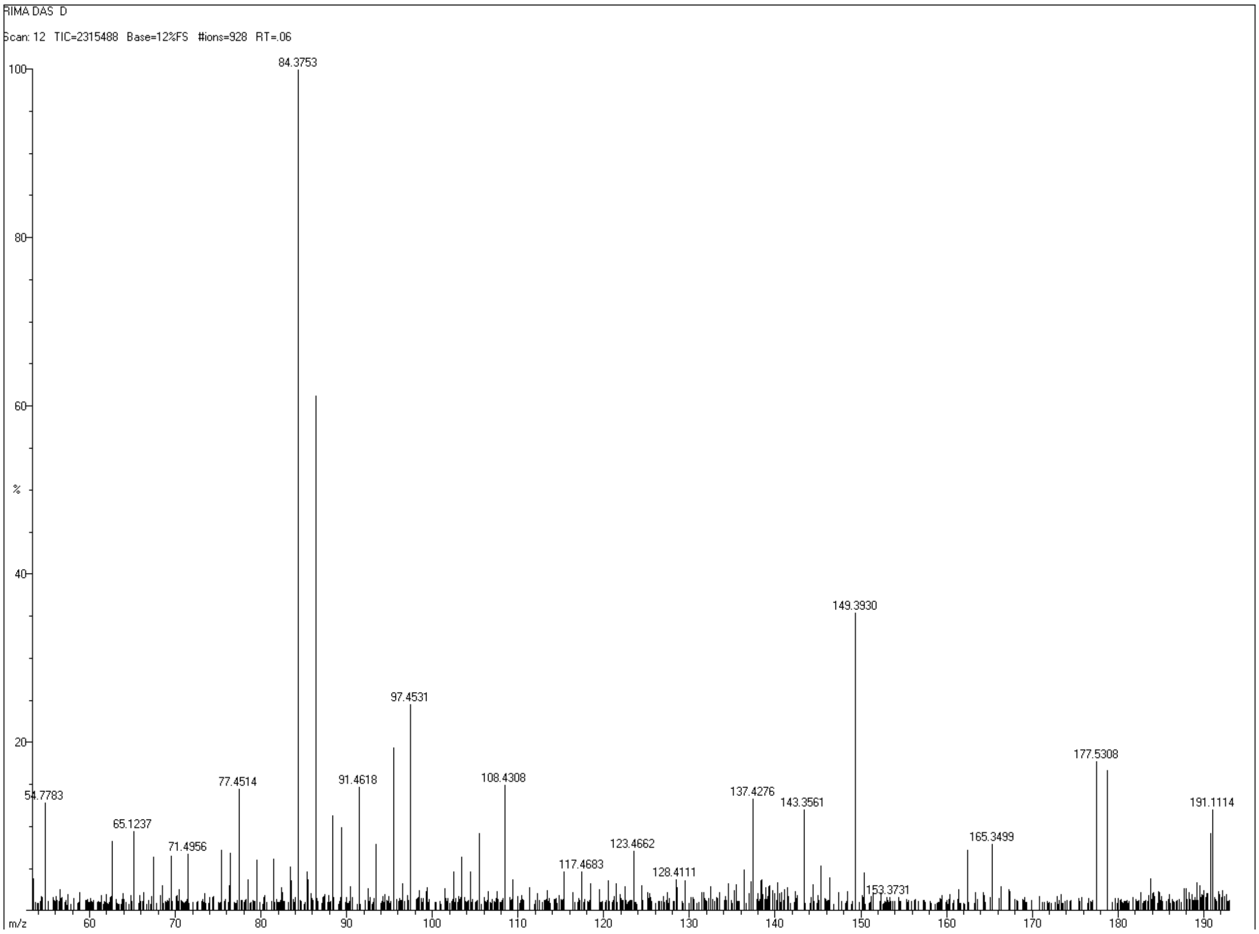

ESI-MS spectrum of 4-methylbiphenyl (Table 1, Entry 2)

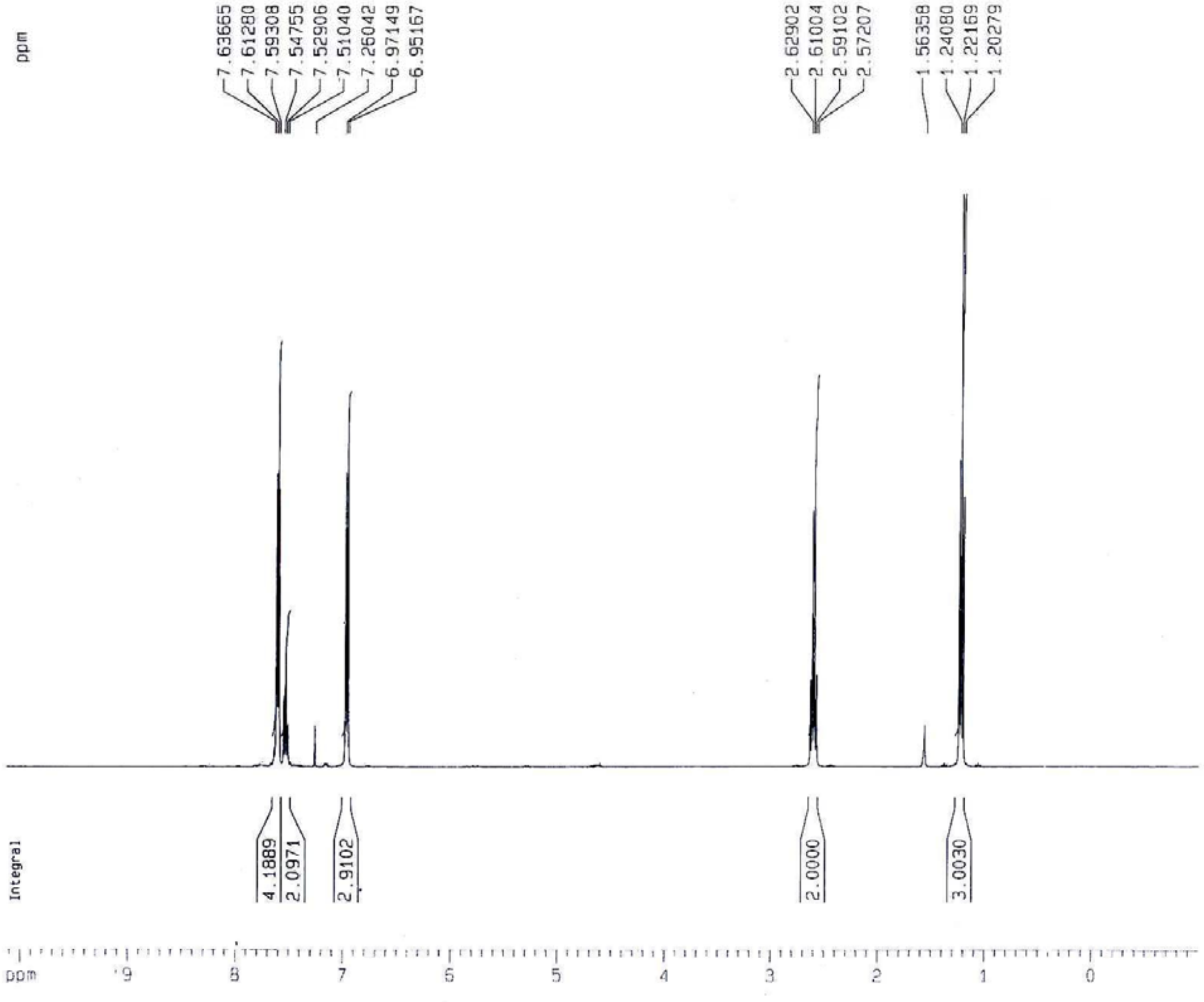

^{1}H-NMR spectrum of 4-ethylbiphenyl (Table 1, Entry 3)

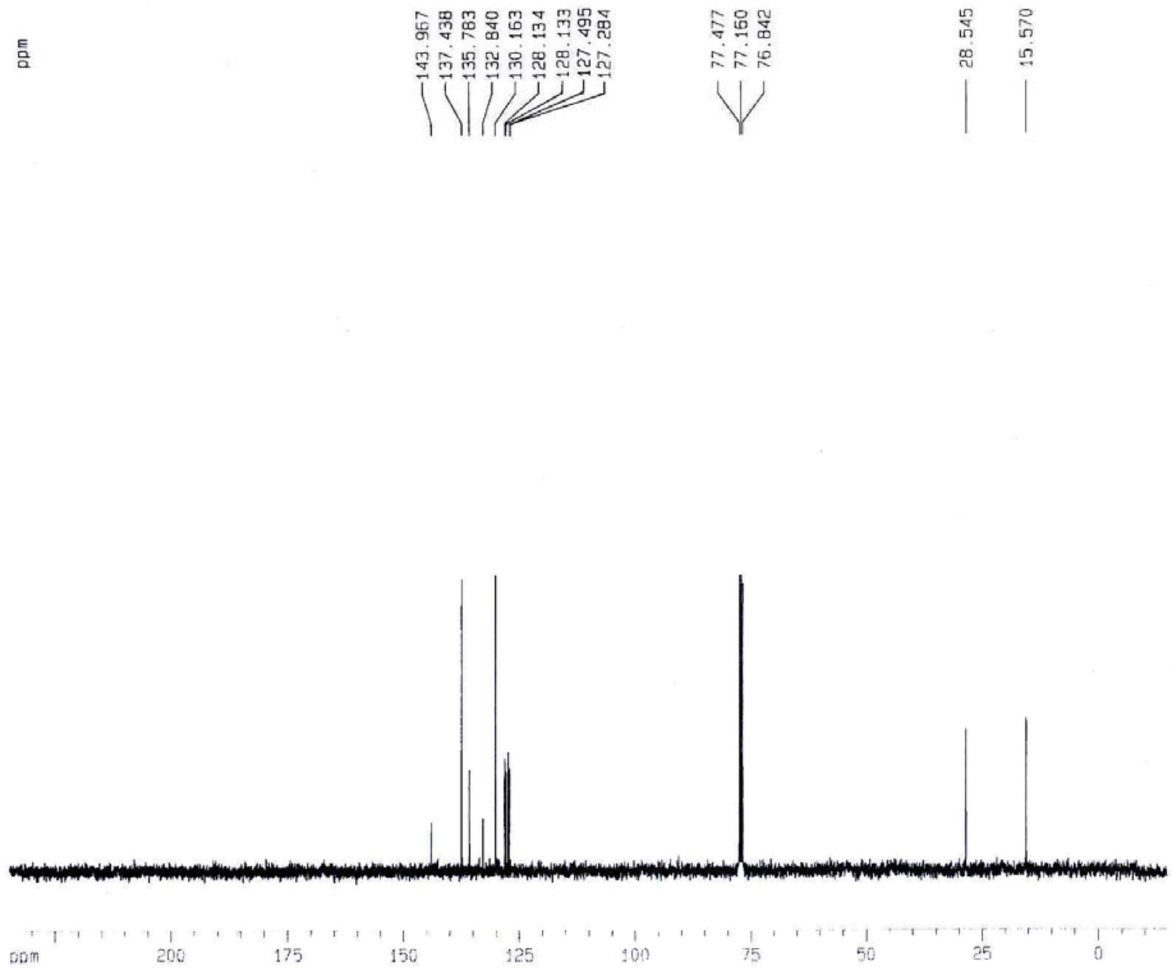

^{13}C-NMR spectrum of 4-ethylbiphenyl (Table 1, Entry 3)

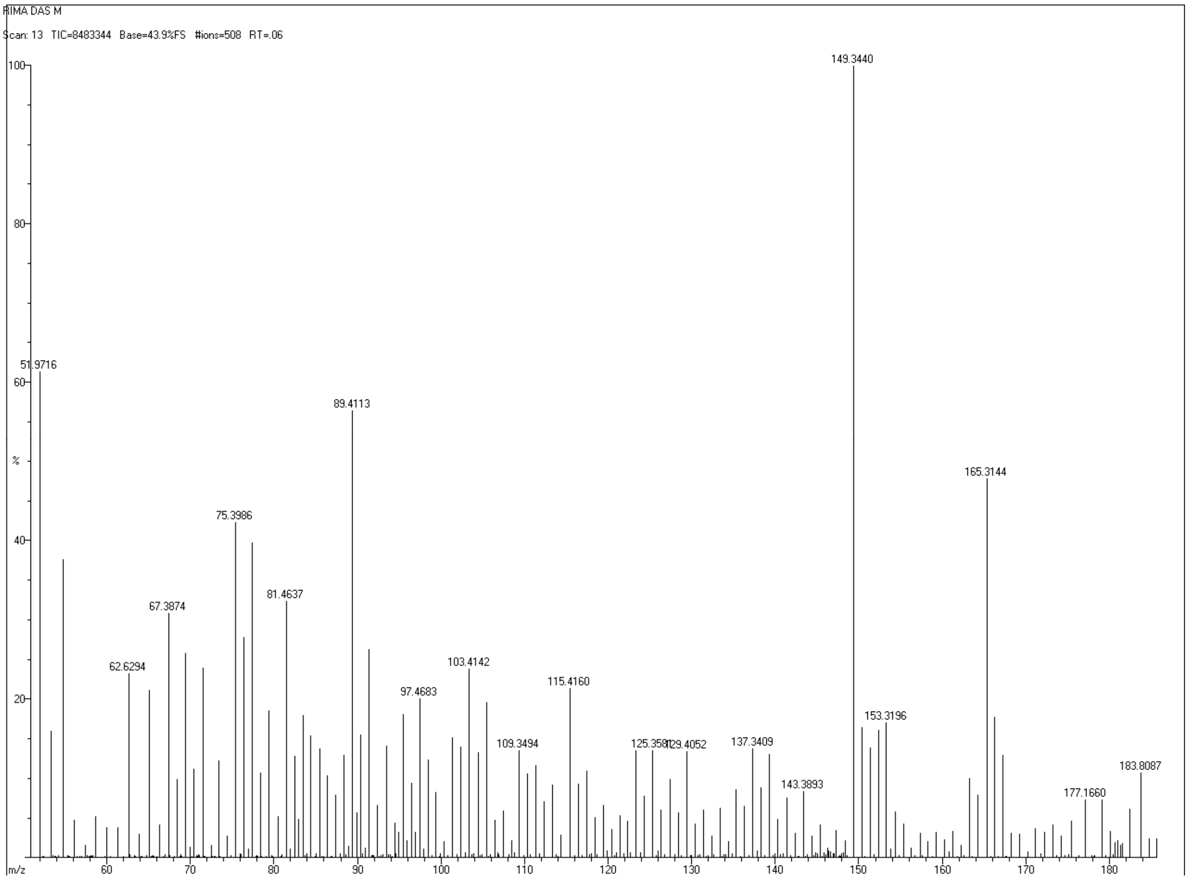

ESI-MS spectrum of 4-ethylbiphenyl (Table 1, Entry 3)

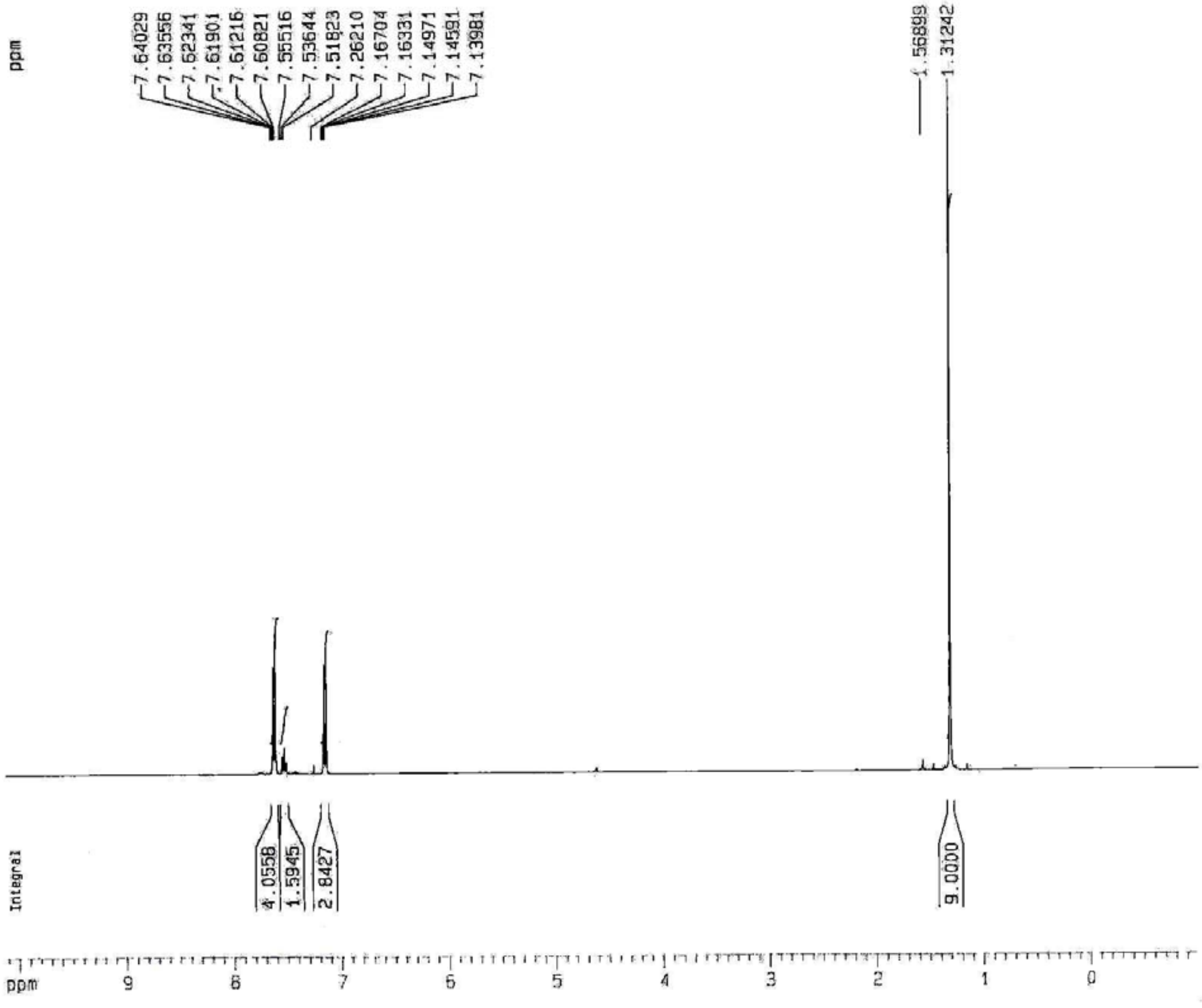

^{1}H-NMR spectrum of 4-[t]butylbiphenyl (Table 1, Entry 4)

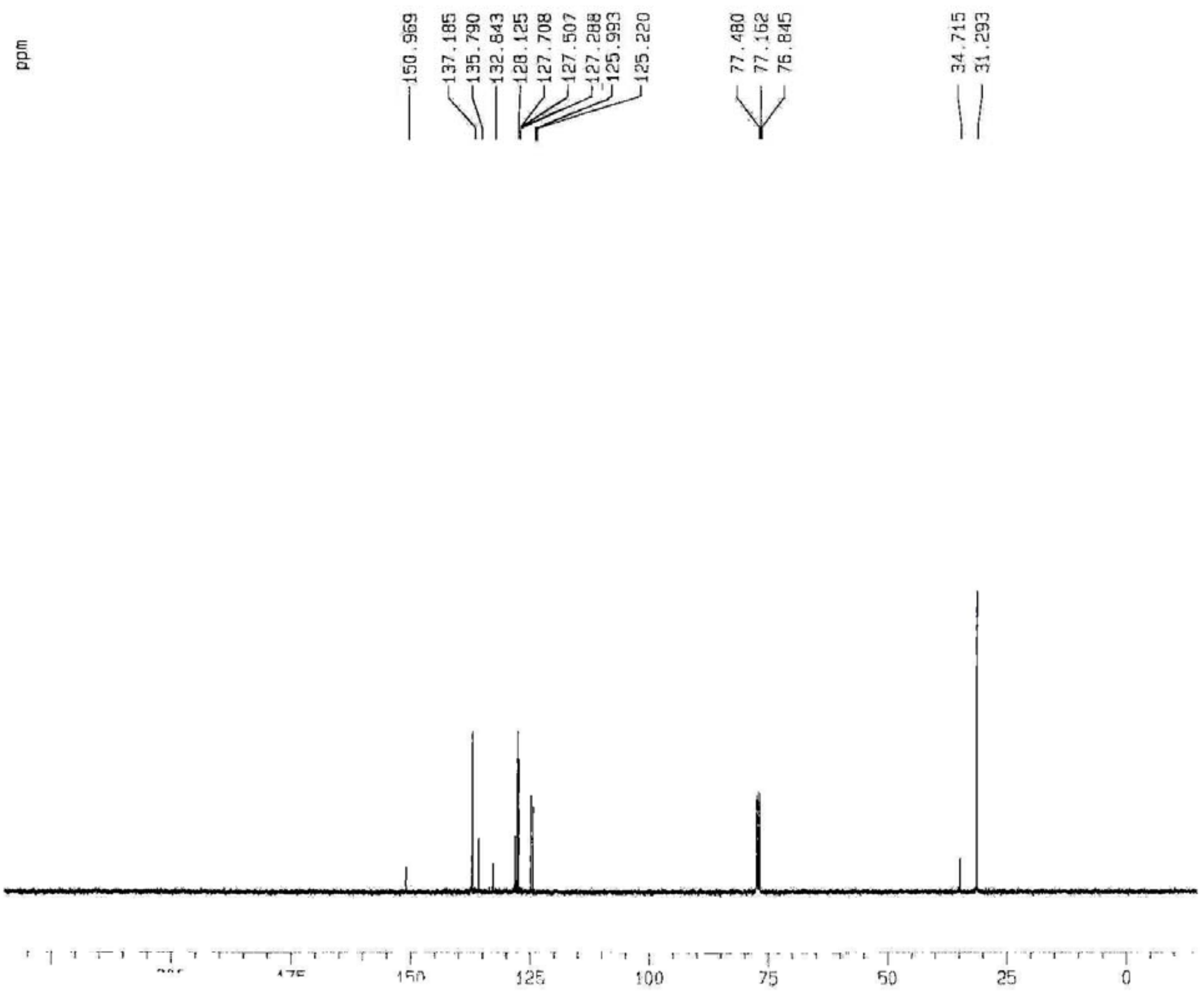

^{13}C-NMR spectrum of 4-^{t}butylbiphenyl (Table 1, Entry 4)

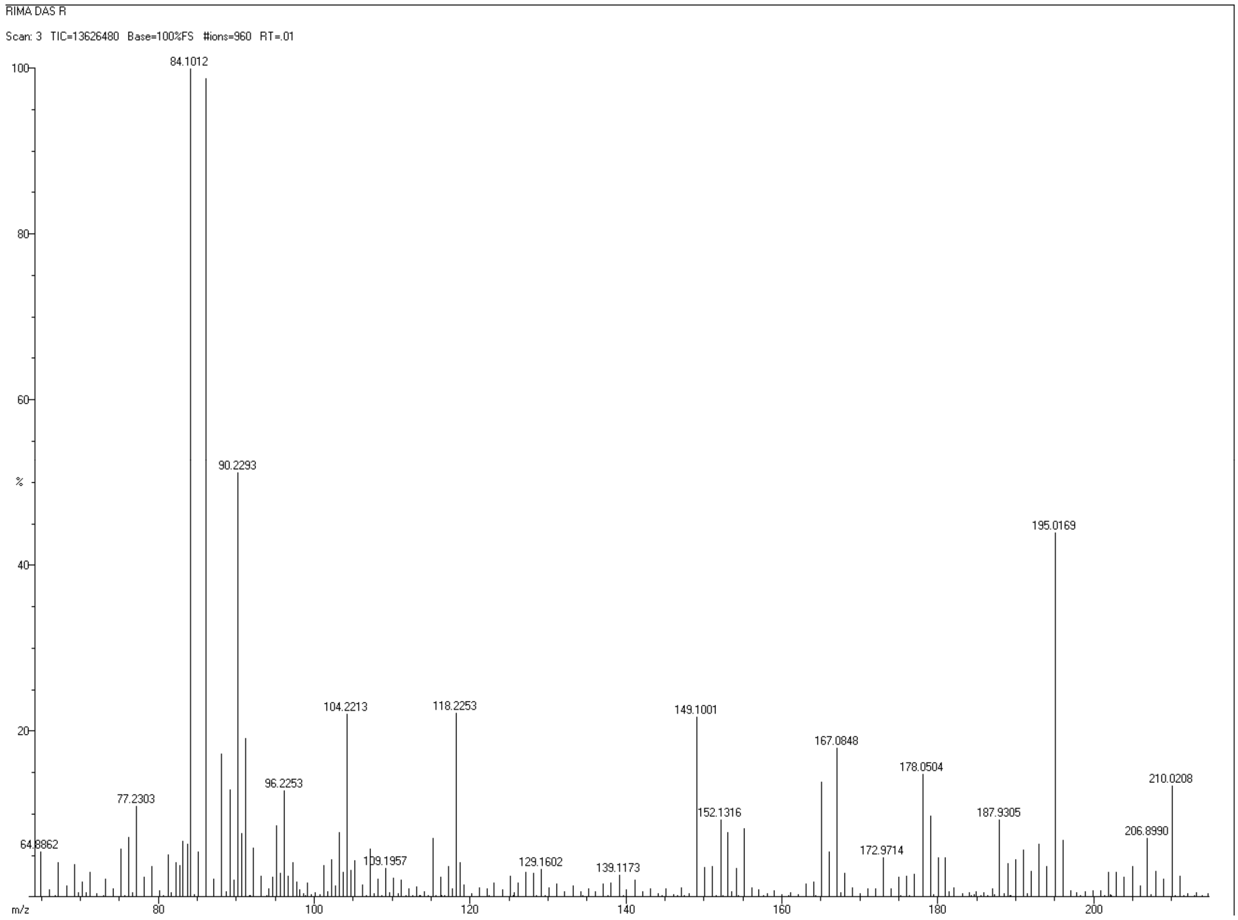

ESI-MS spectrum of 4-^{t}butylbiphenyl (Table 1, Entry 4)

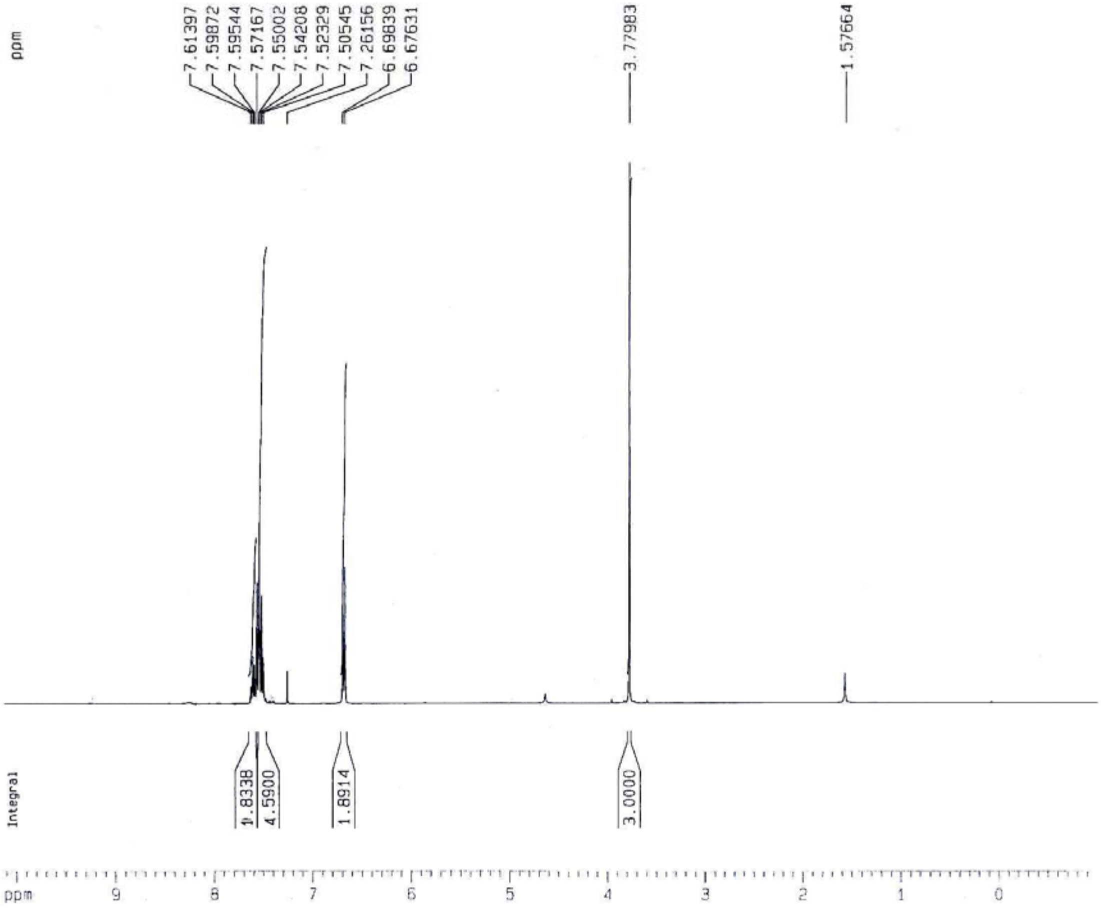

^{1}H-NMR spectrum of 4-methoxybiphenyl (Table 1, Entry 6)

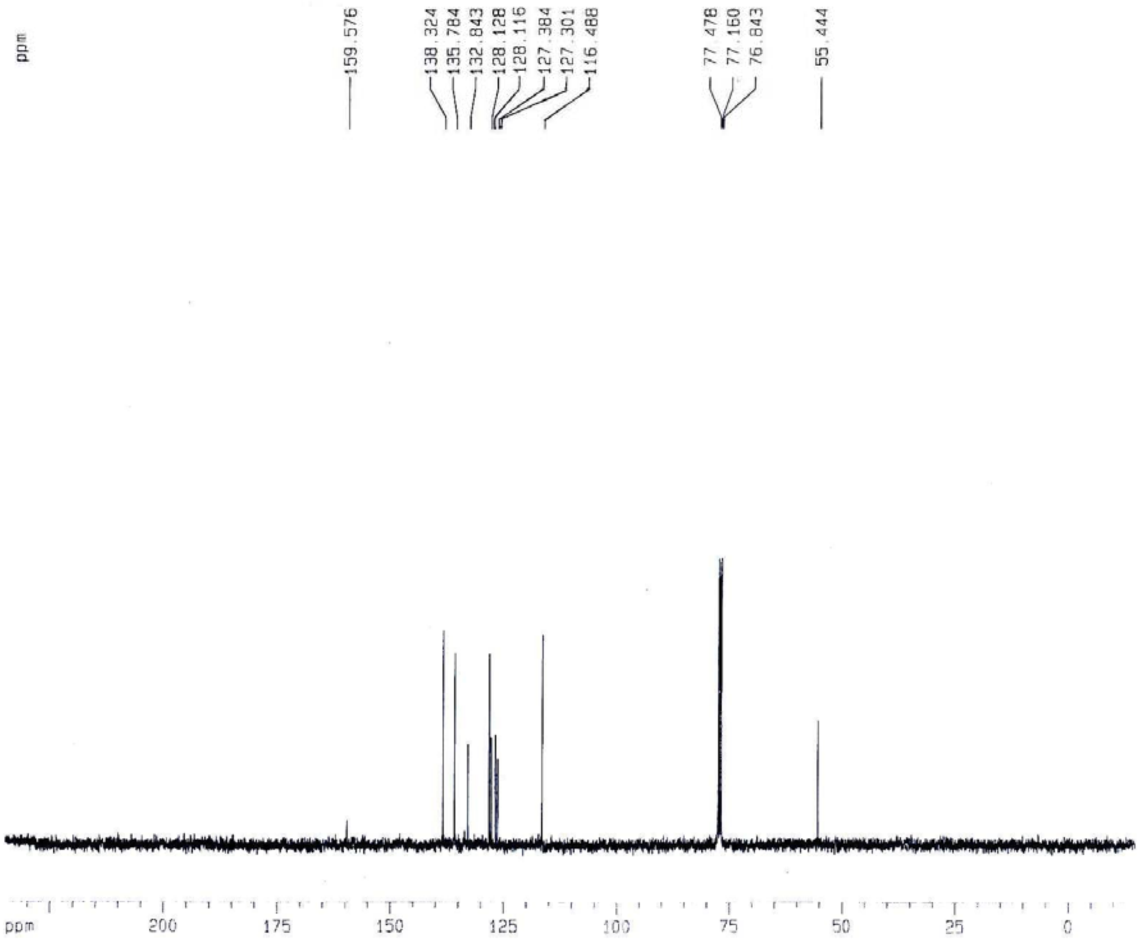

^{13}C-NMR spectrum of 4-methoxybiphenyl (Table 1, Entry 6)

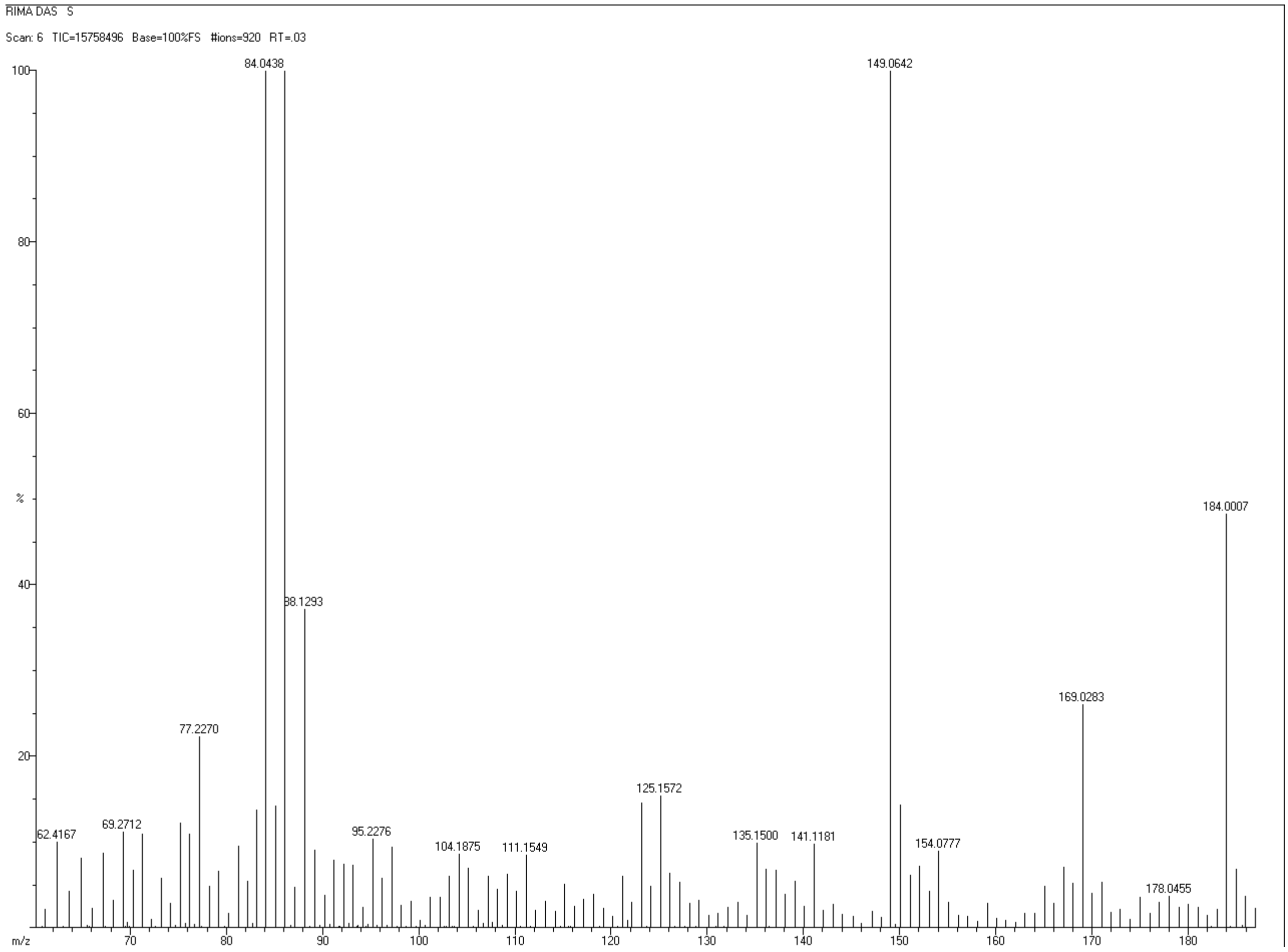

ESI-MS spectrum of 4-methoxybiphenyl (Table 1, Entry 6)

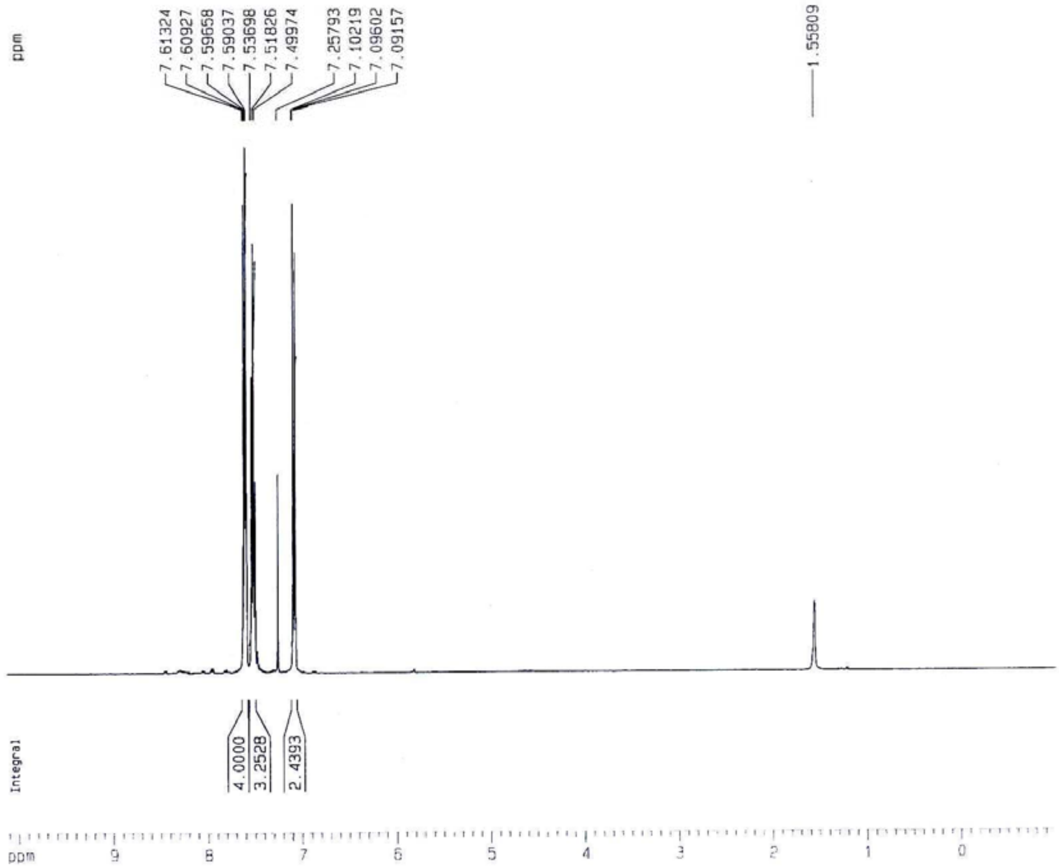

^{1}H-NMR spectrum of 4-chlorobiphenyl (Table 1, Entry 7)

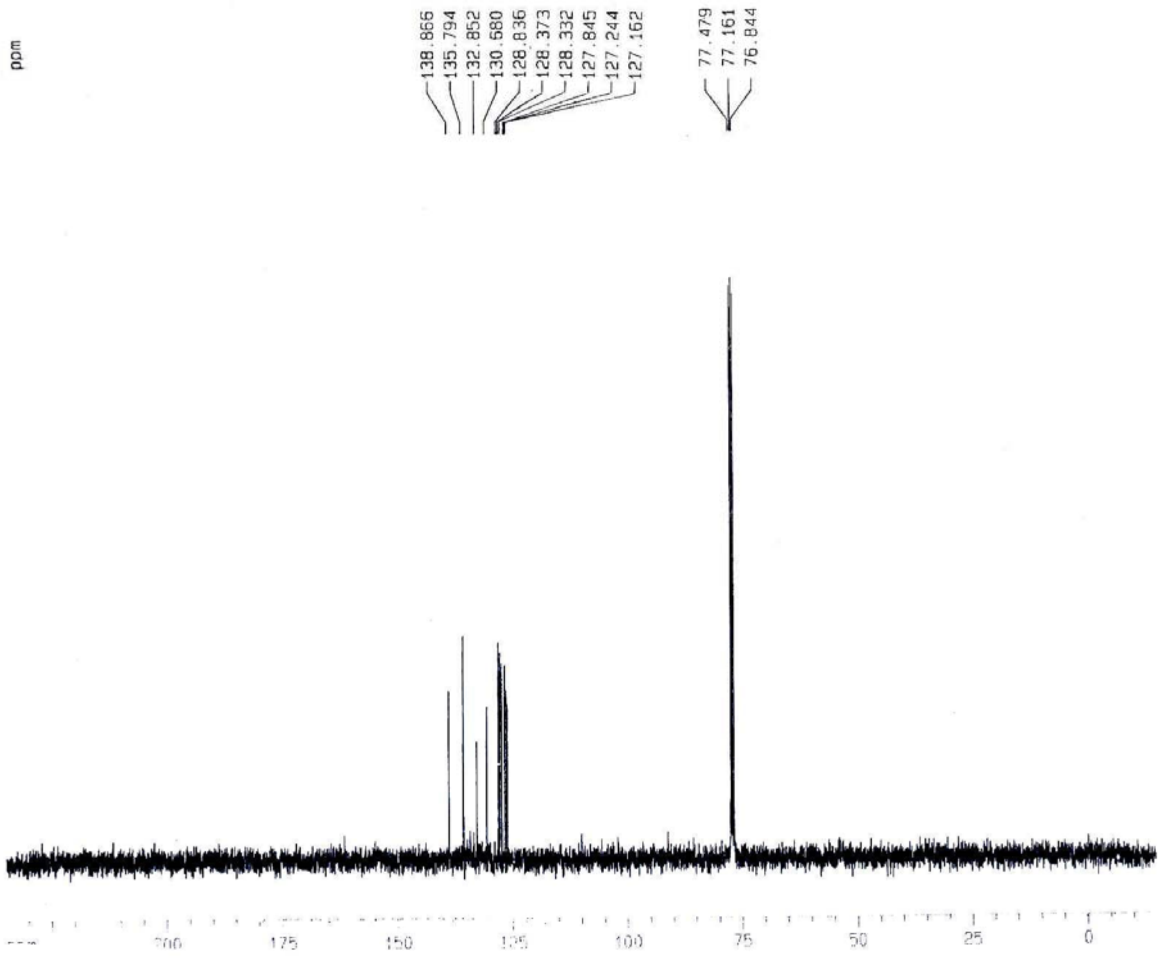

^{13}C-NMR spectrum of 4-chlorobiphenyl (Table 1, Entry 7)

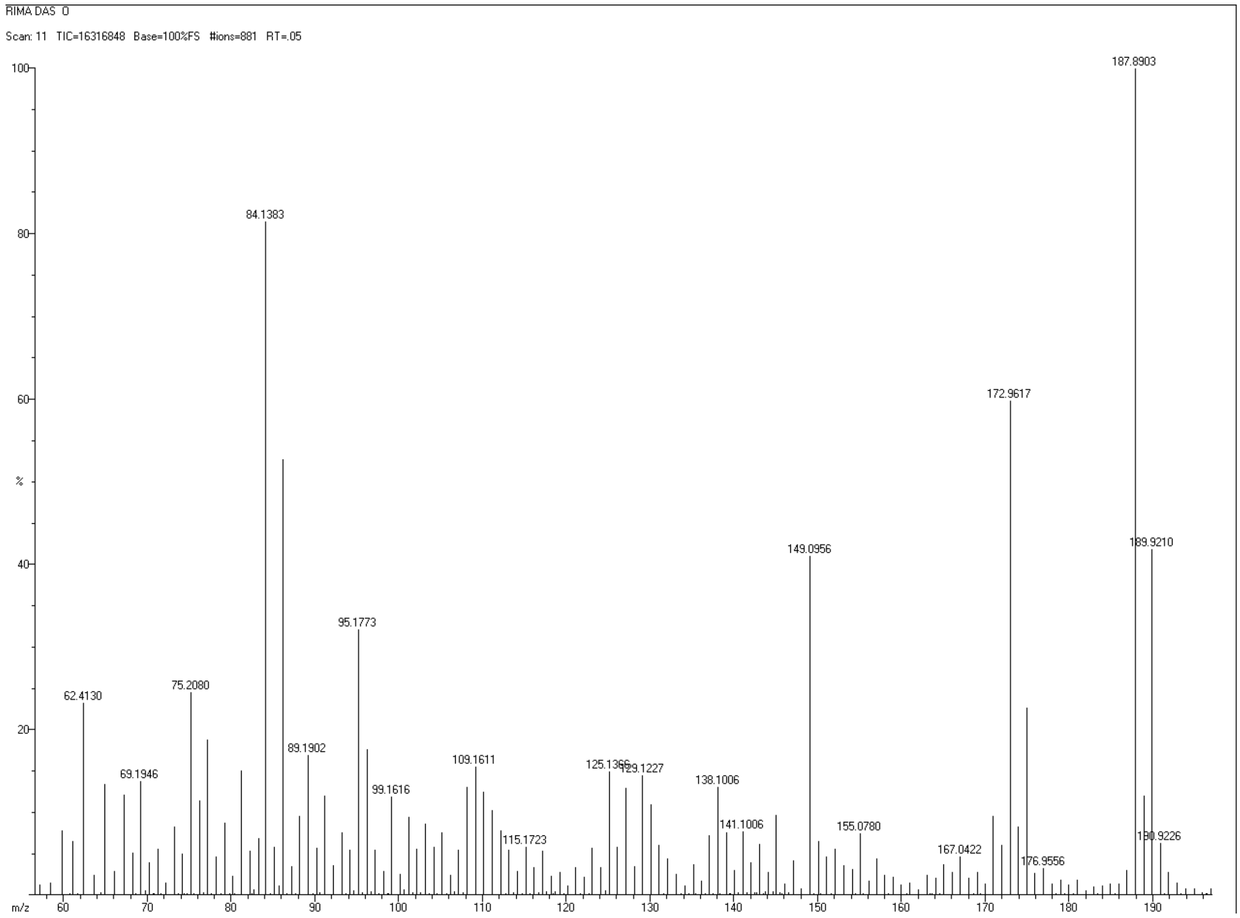

ESI-MS spectrum of 4-chlorobiphenyl (Table 1, Entry 7)

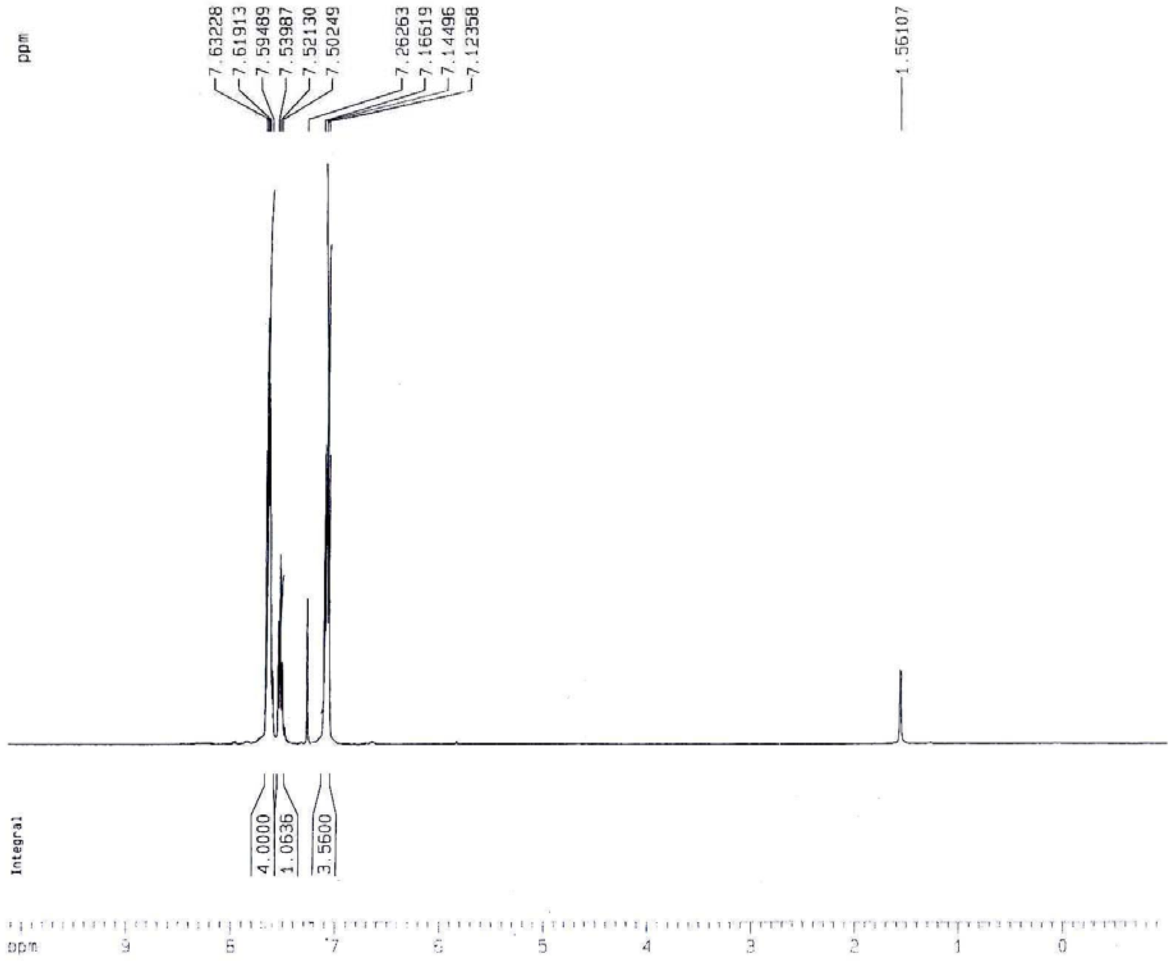

^{1}H-NMR spectrum of 4-fluorobiphenyl (Table 1, Entry 8)

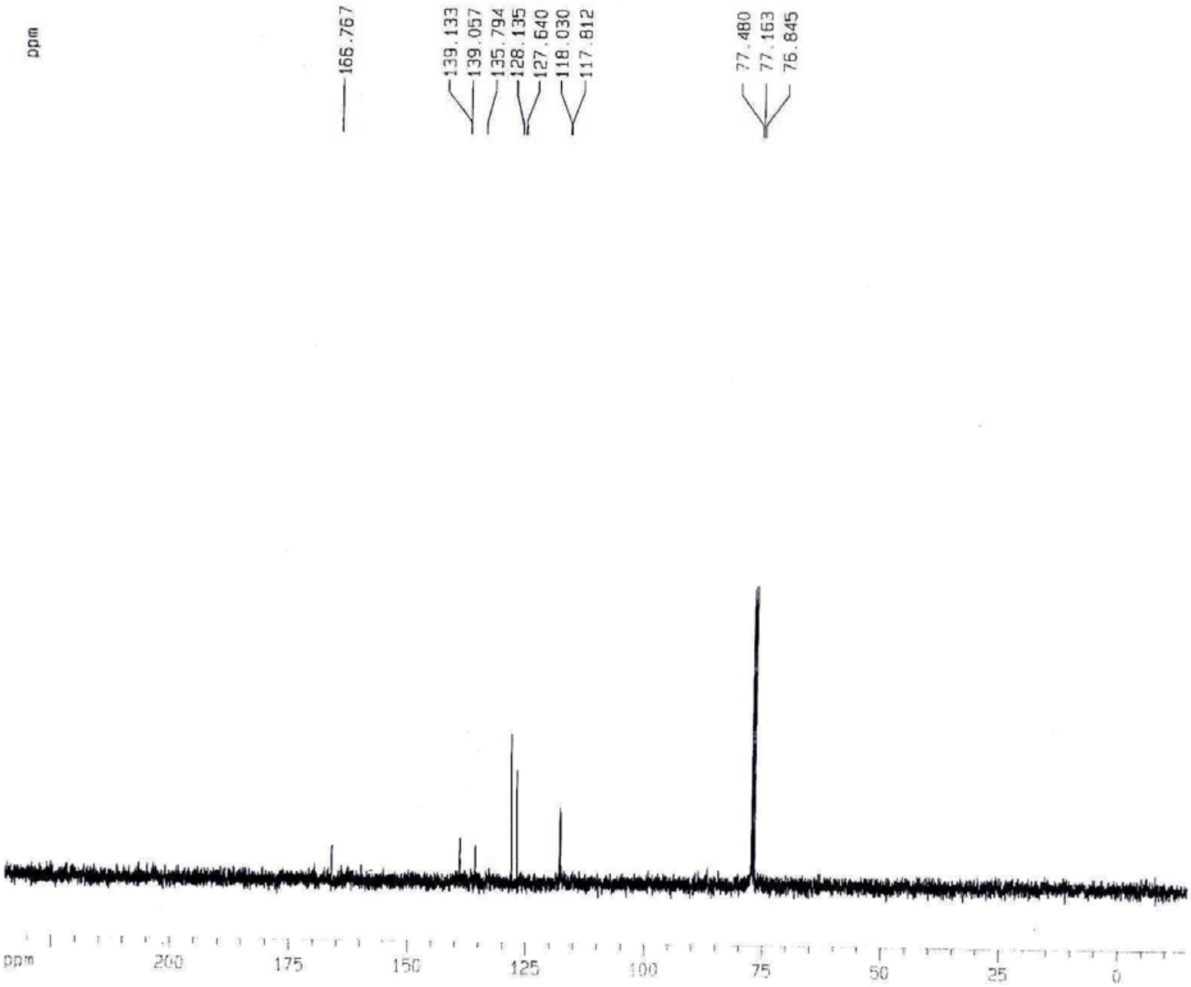

^{13}C-NMR spectrum of 4-fluorobiphenyl (Table 1, Entry 8)

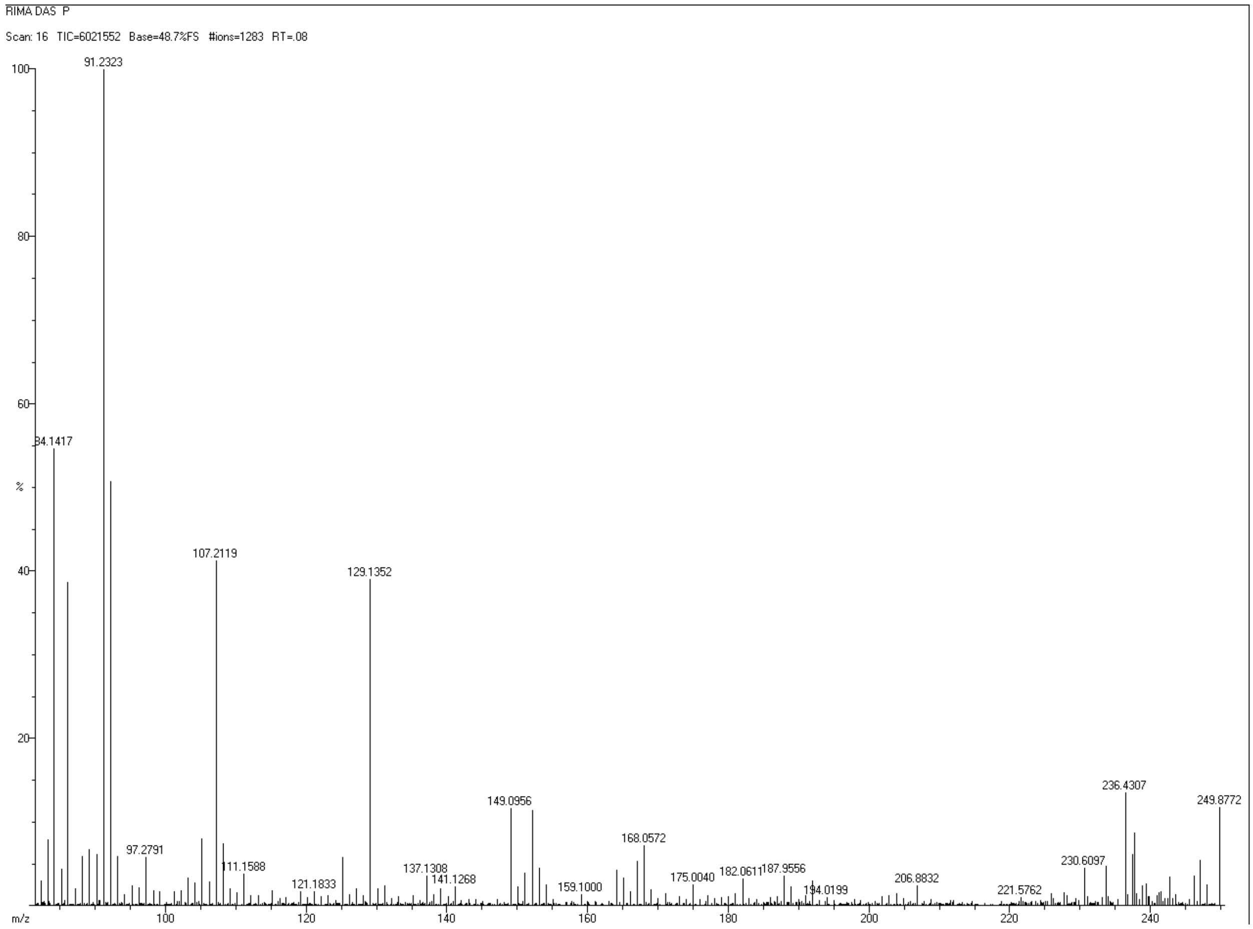

ESI-MS spectrum of 4-fluorobiphenyl (Table 1, Entry 8)

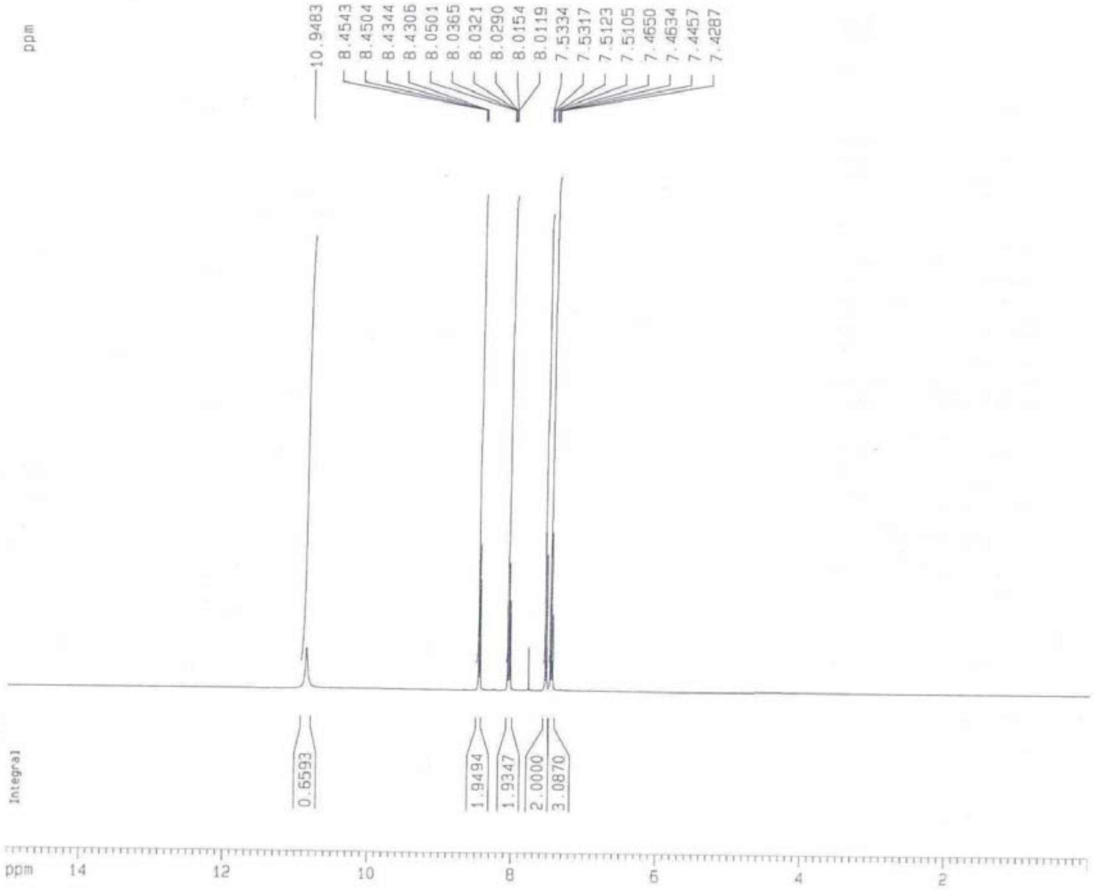

^{1}H-NMR spectrum of Biphenyl-4-carboxylic acid (Table 1, Entry 10)

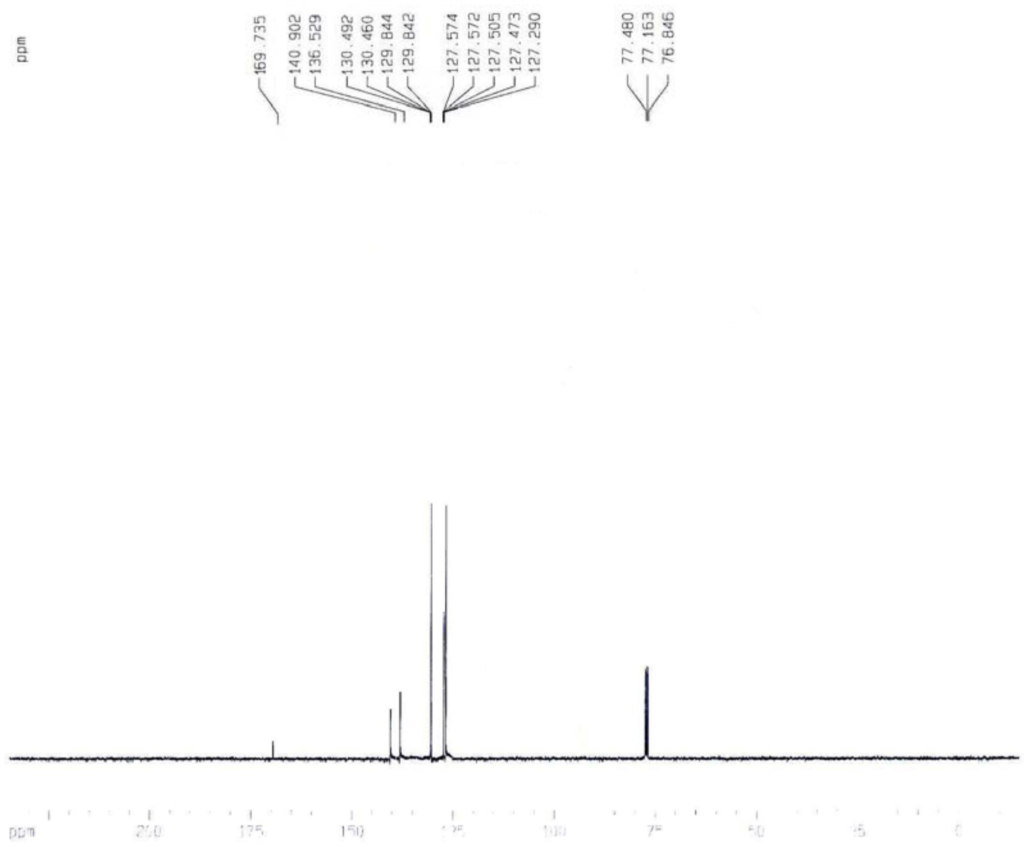

^{13}C-NMR spectrum of Biphenyl-4-carboxylic acid (Table 1, Entry 10)

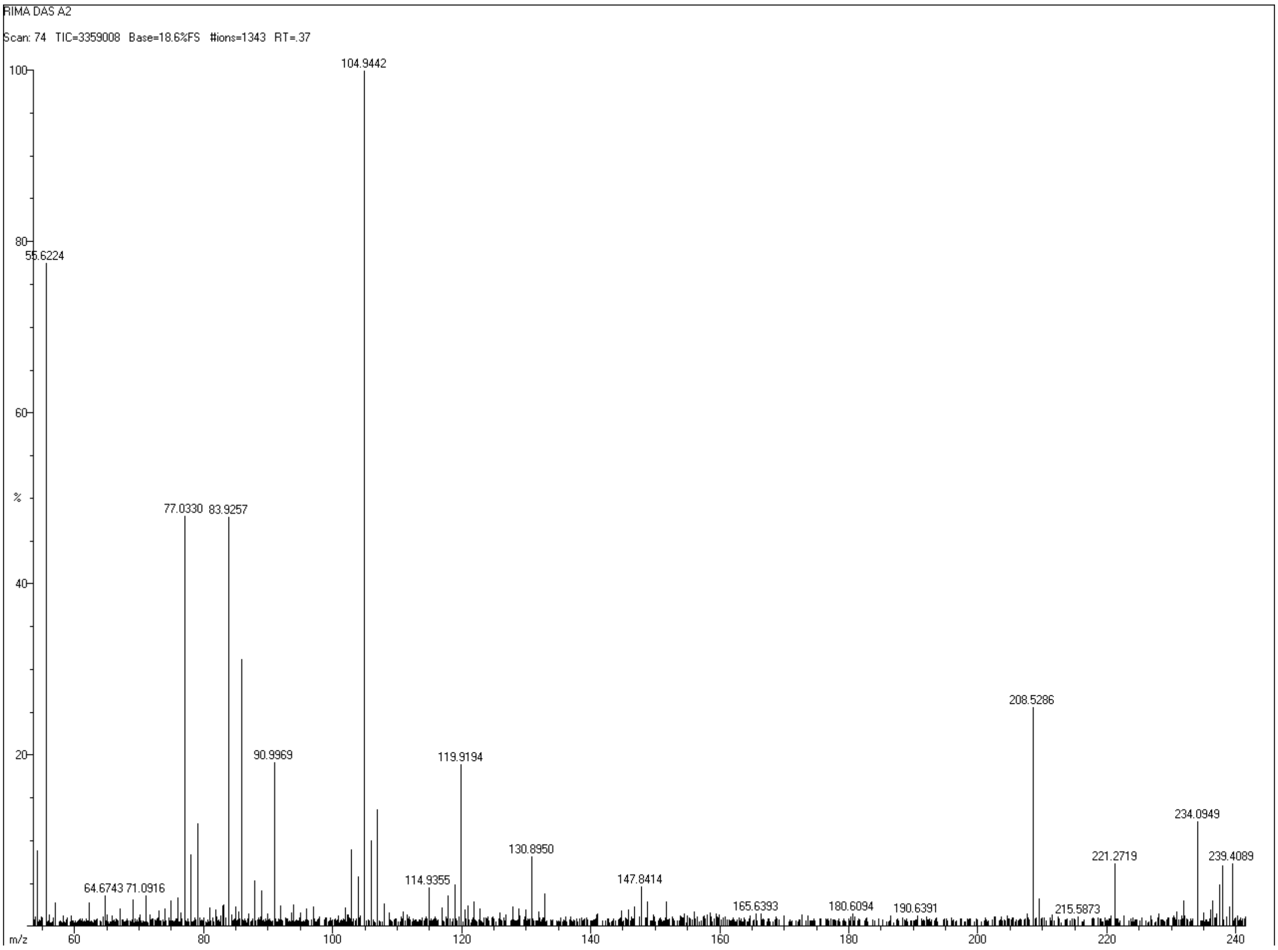

ESI-MS spectrum of Biphenyl-4-carboxylic acid (Table 1, Entry 10)

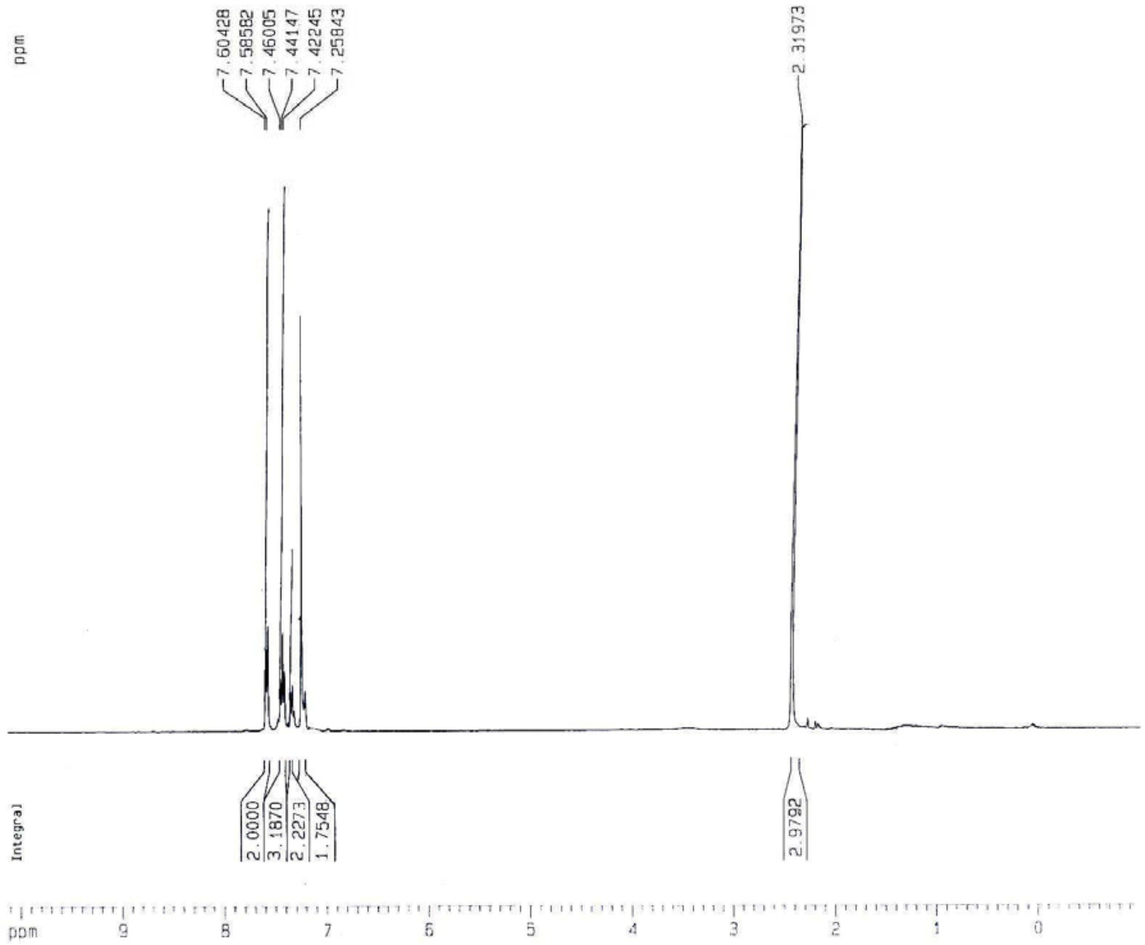

^{1}H-NMR spectrum of 4-methylbiphenyl (Table 2, Entry 1)

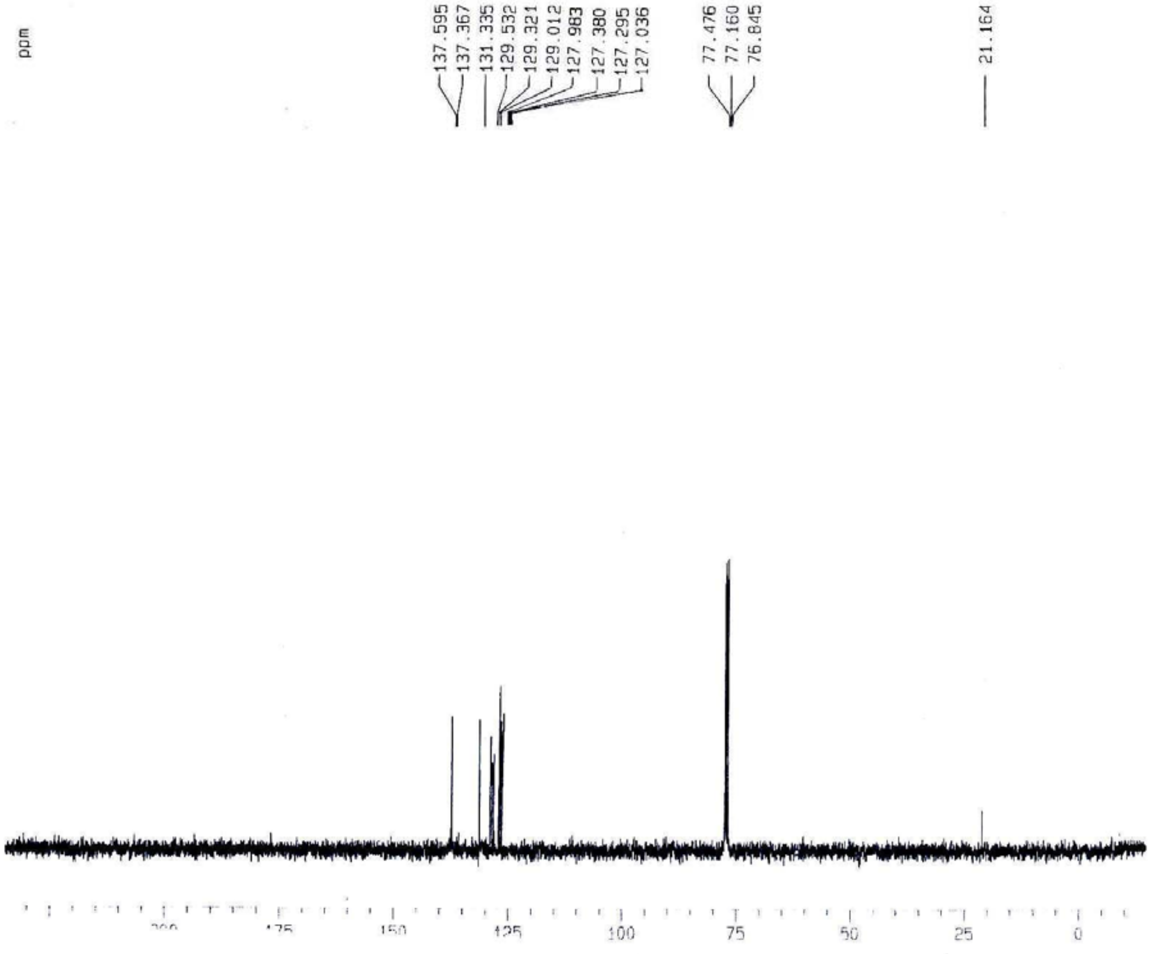

^{13}C-NMR spectrum of 4-methylbiphenyl (Table 2, Entry 1)

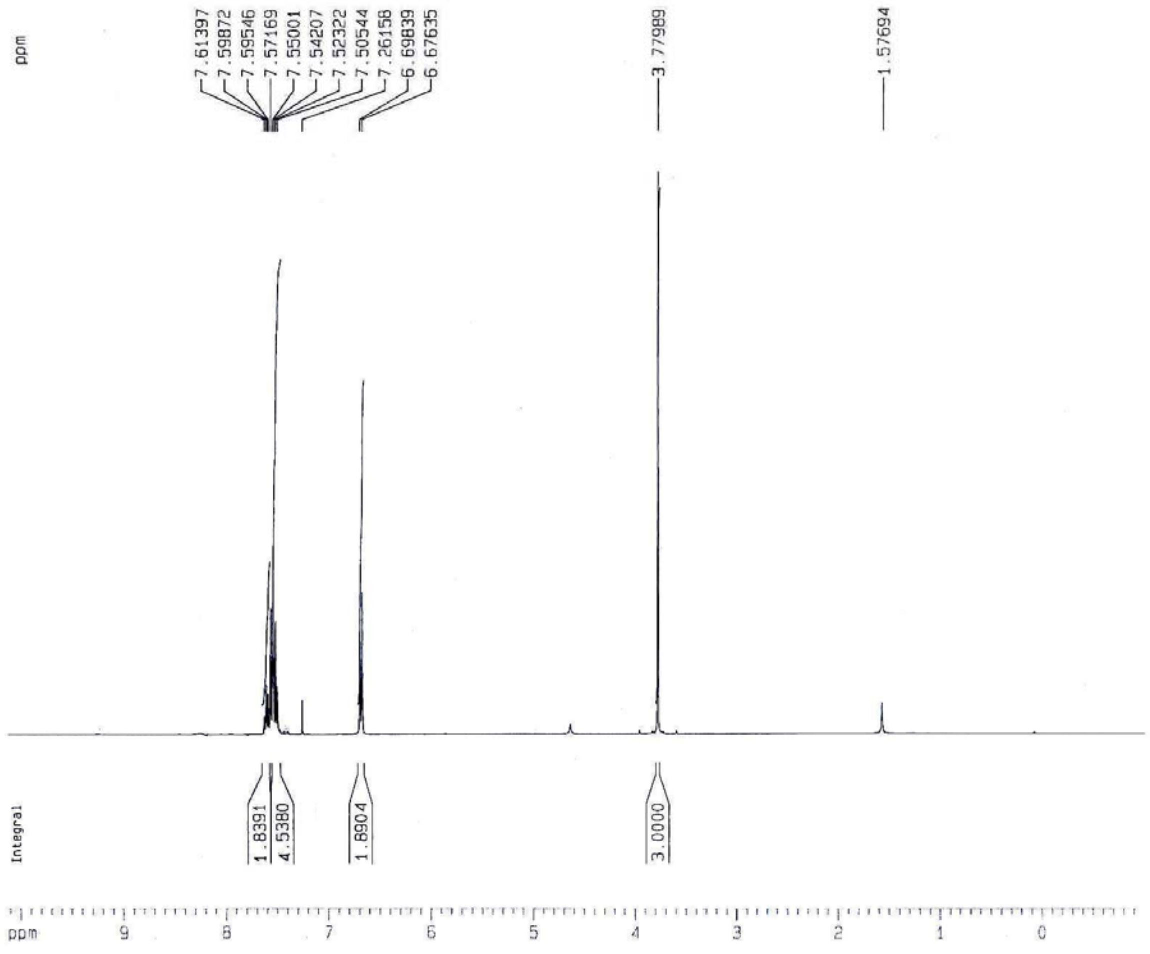

^{1}H-NMR spectrum of 4-methoxybiphenyl (Table 2, Entry 2)

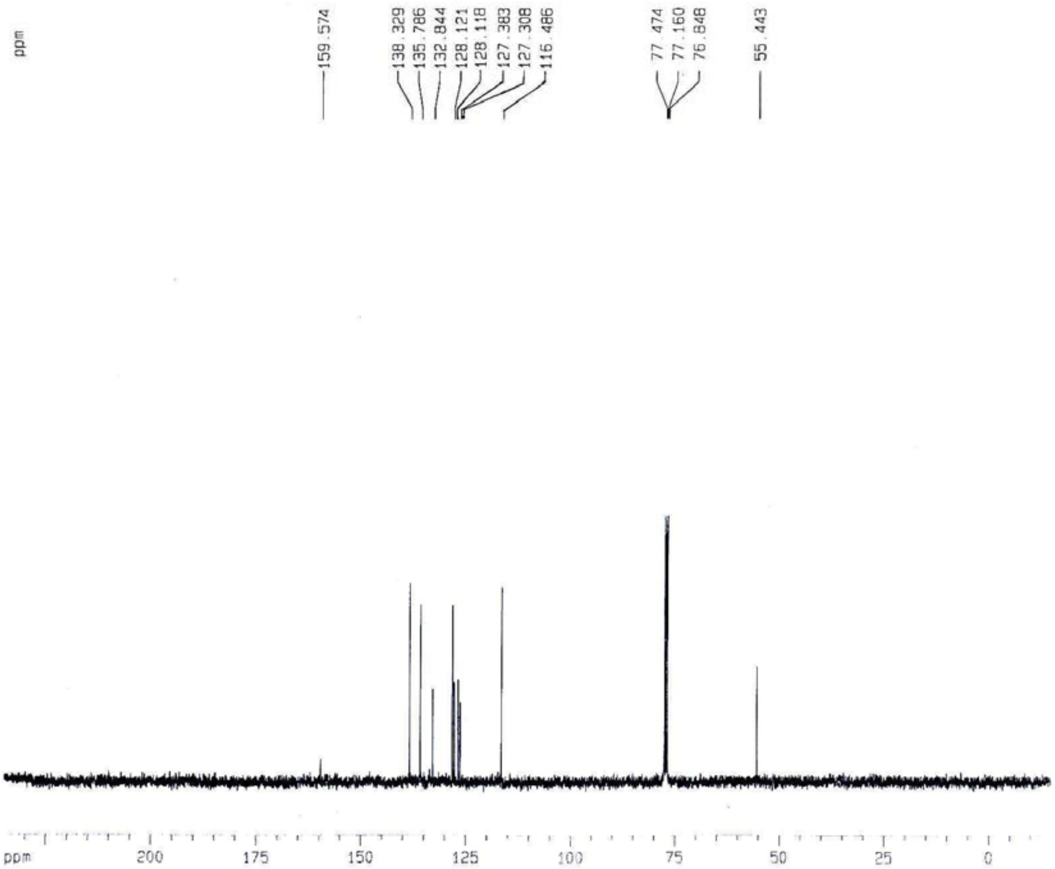

^{13}C-NMR spectrum of 4-methoxybiphenyl (Table 2, Entry 2)

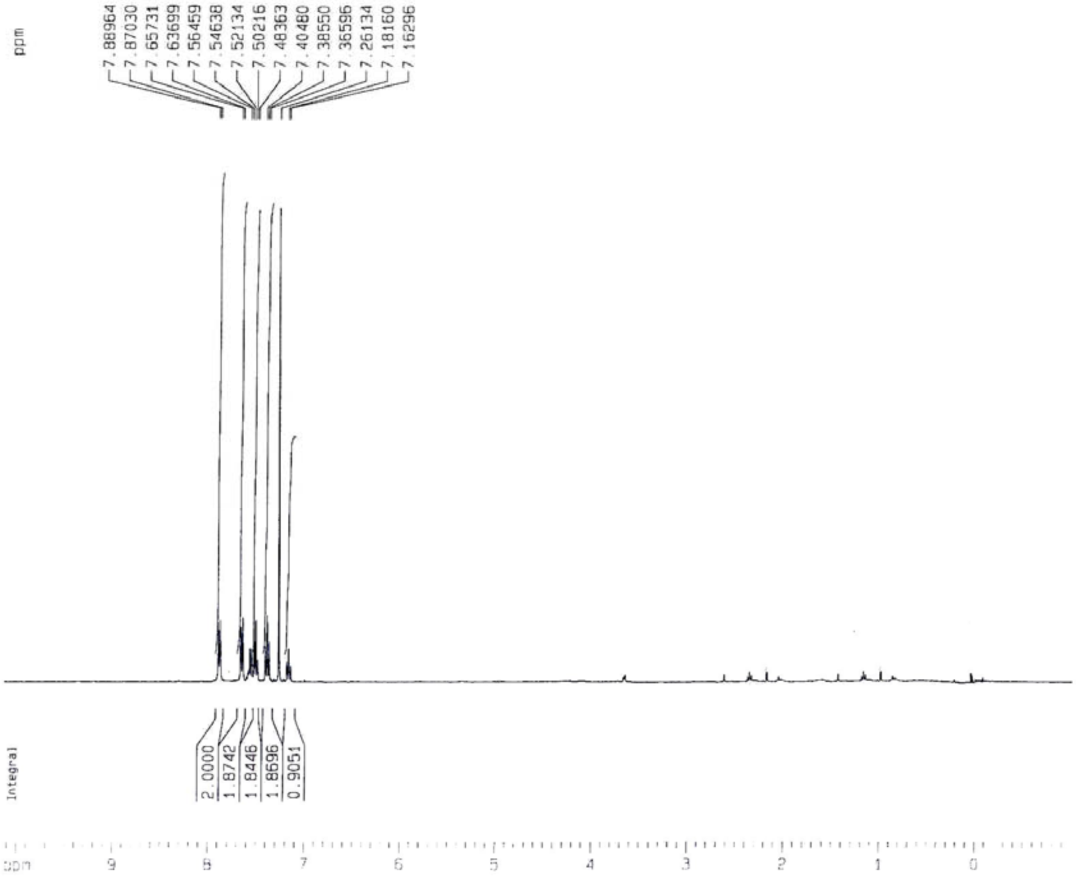

^{1}H-NMR spectrum of 4-cyanobiphenyl (Table 2, Entry 3)

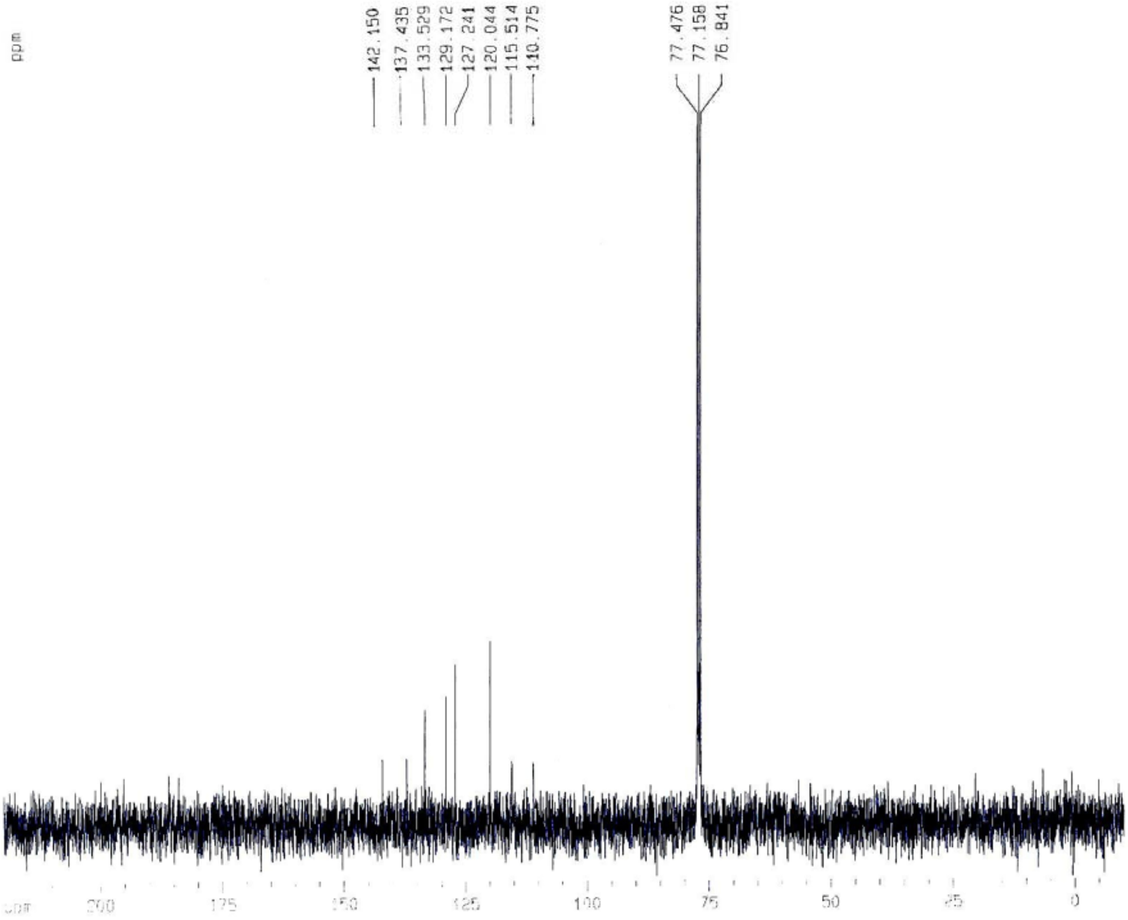

^{13}C-NMR spectrum of 4-cyanobiphenyl (Table 2, Entry 3)

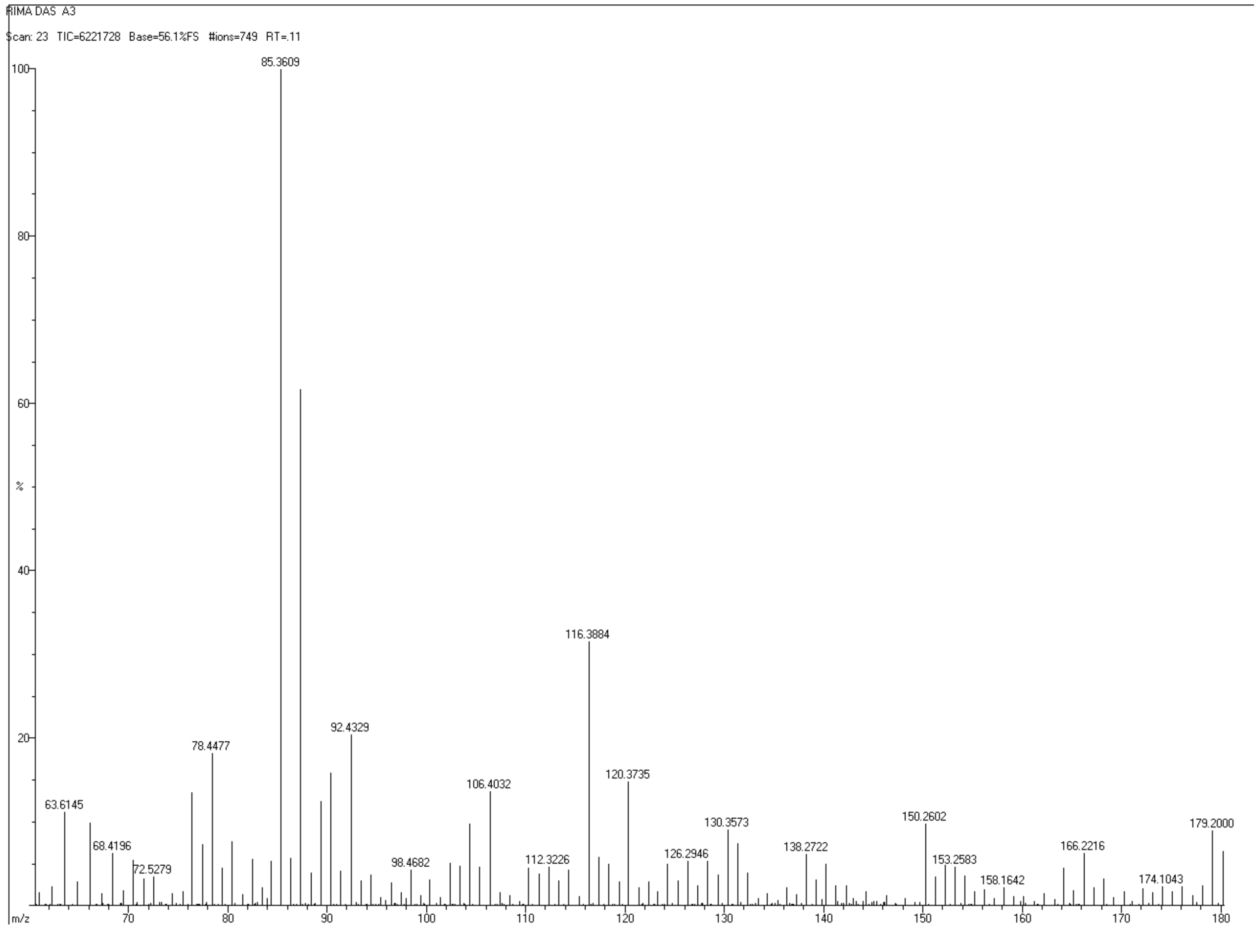

ESI-MS spectrum of 4-cyanobiphenyl (Table 2, Entry 3)

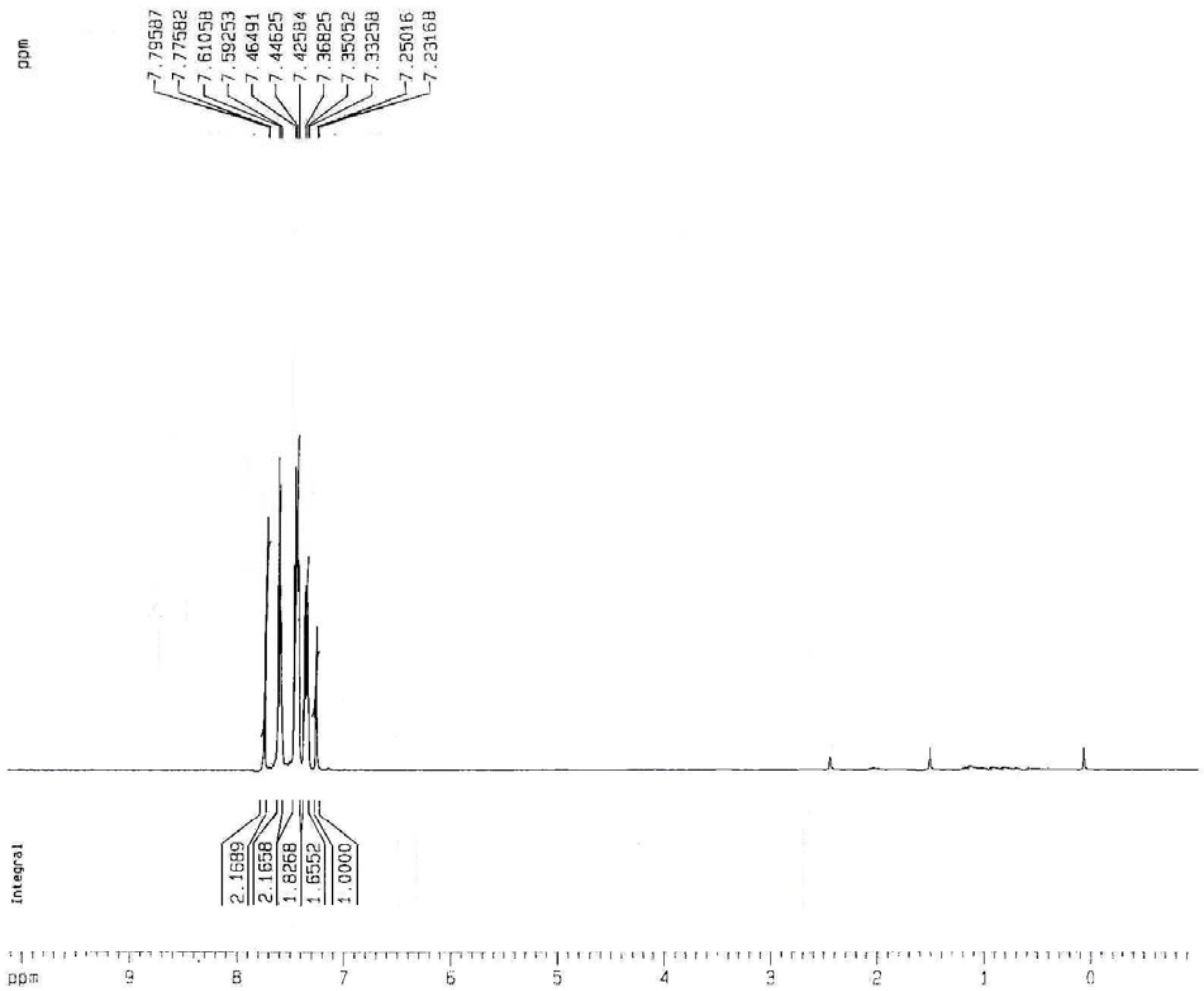

^{1}H-NMR spectrum of 4-trifluoromethylbiphenyl (Table 2, Entry 4)

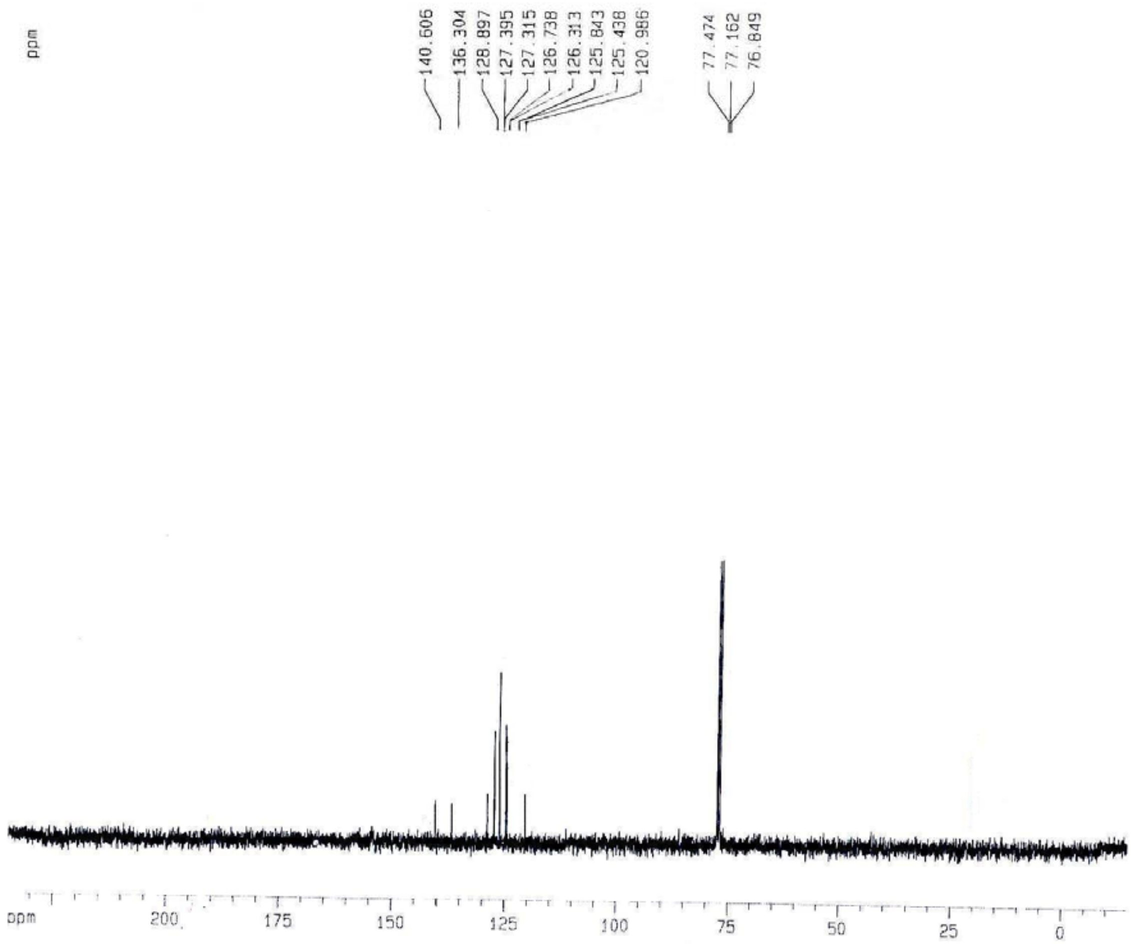

^{13}C-NMR spectrum of 4-trifluoromethylbiphenyl (Table 2, Entry 4)

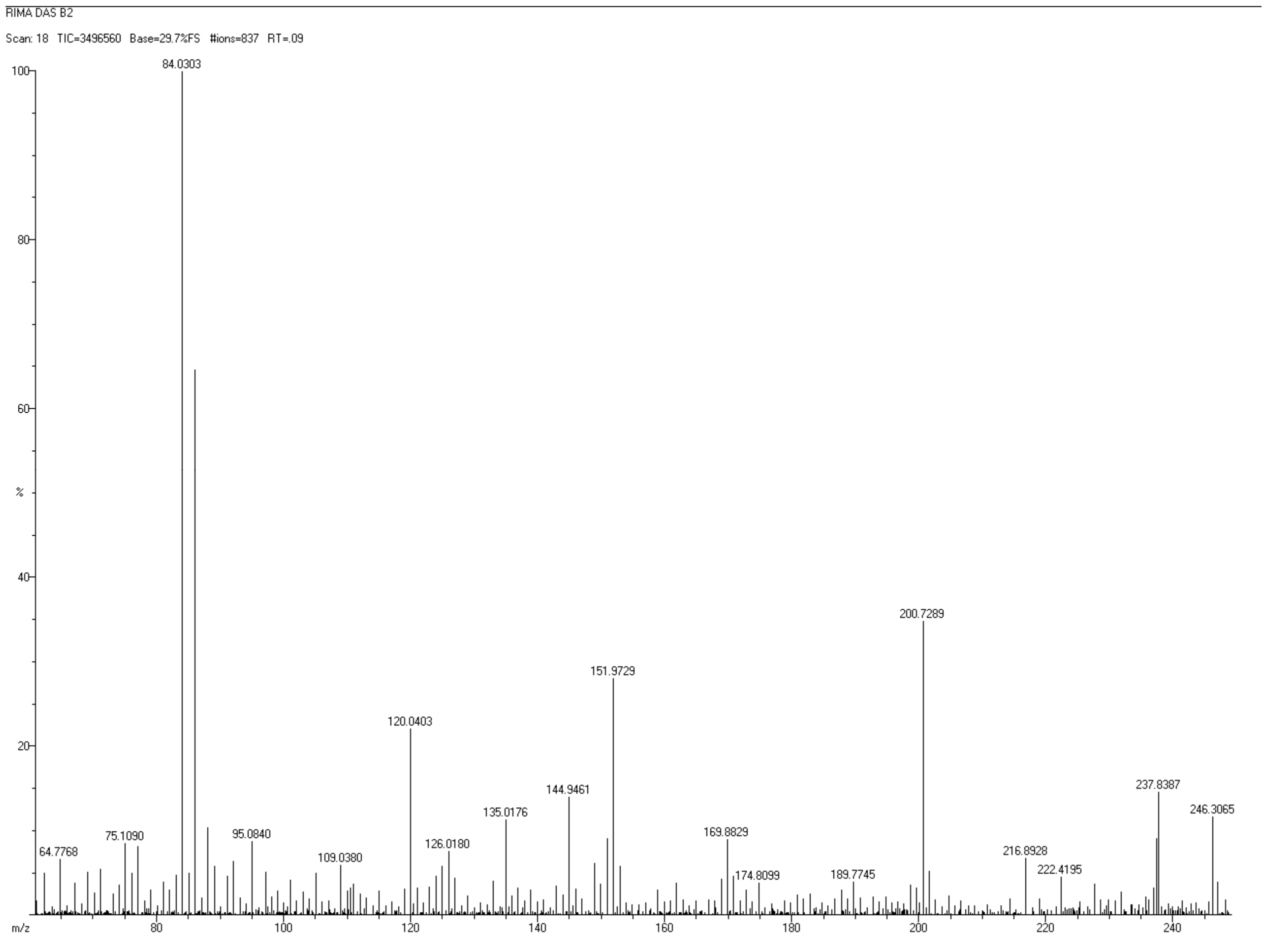

ESI-MS spectrum of 4-trifluoromethylbiphenyl (Table 2, Entry 4)

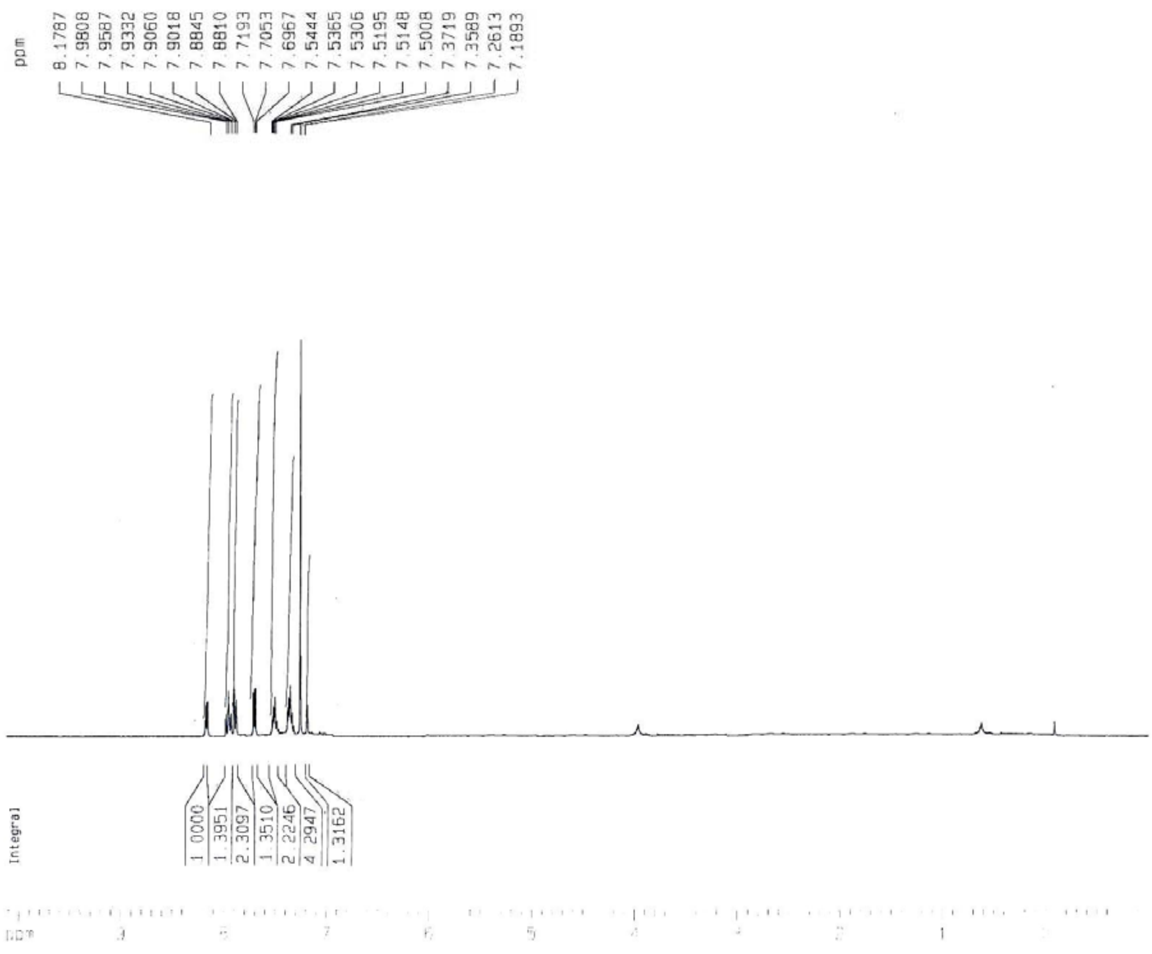

^{1}H-NMR spectrum of 2-phenylnapthylene (Table 2, Entry 5)

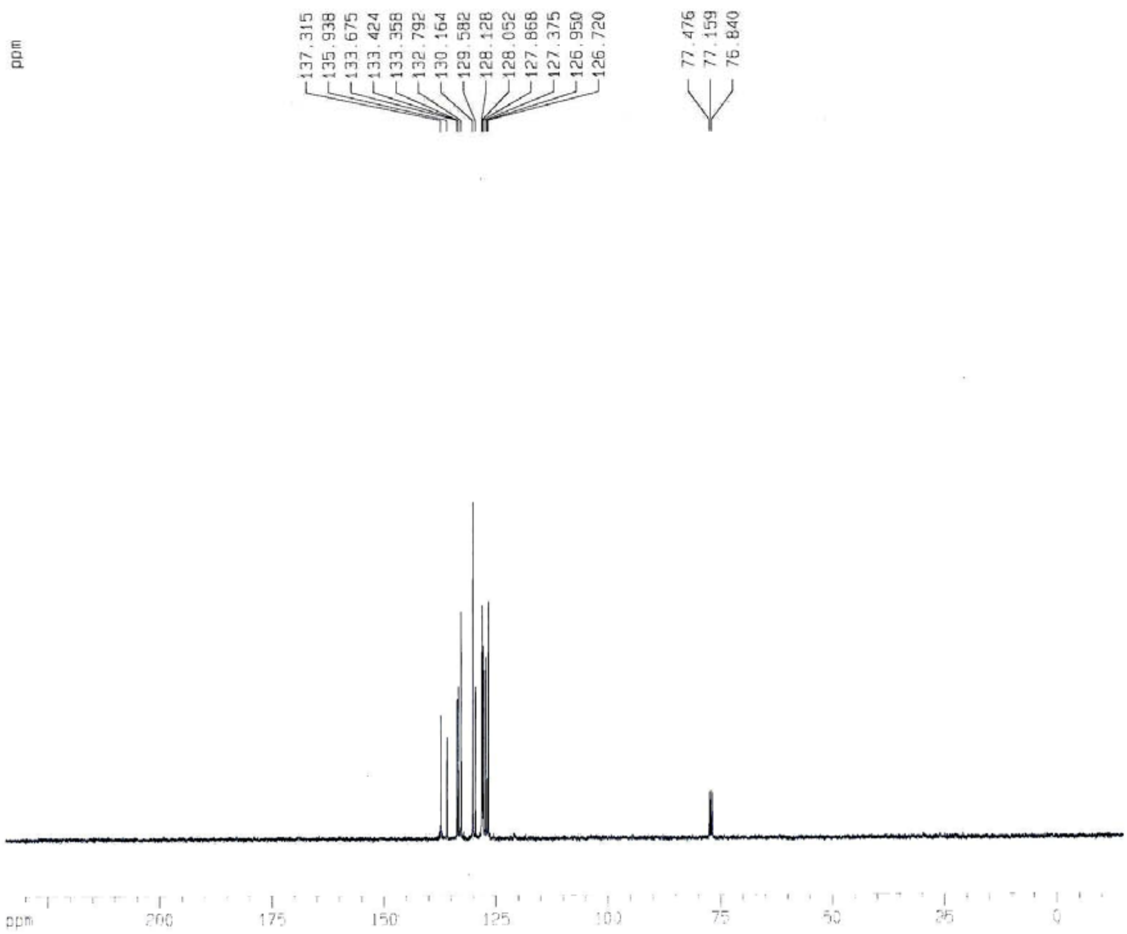

^{13}C-NMR spectrum of 2-phenylnapthylene (Table 2, Entry 5)

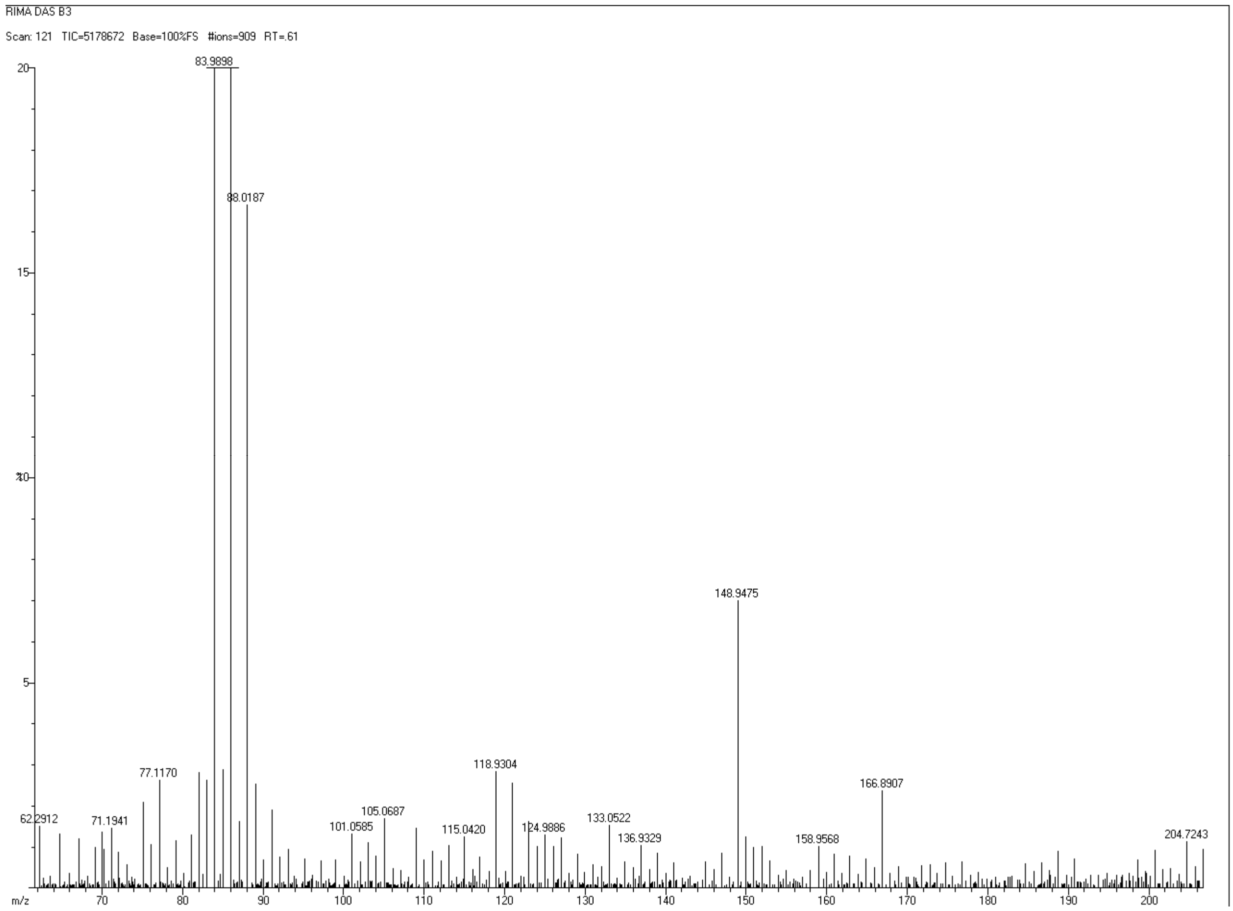

ESI-MS spectrum of 2-phenylnapthylene (Table 2, Entry 5)
